# Diagnostic Profiling of the Human Public IgM Repertoire With Scalable Mimotope Libraries

**DOI:** 10.3389/fimmu.2019.02796

**Published:** 2019-12-03

**Authors:** Anastas Pashov, Velizar Shivarov, Maya Hadzhieva, Victor Kostov, Dilyan Ferdinandov, Karen-Marie Heintz, Shina Pashova, Milena Todorova, Tchavdar Vassilev, Thomas Kieber-Emmons, Leonardo A. Meza-Zepeda, Eivind Hovig

**Affiliations:** ^1^Laboratory of Experimental Immunotherapy, Department of Immunology, Stephan Angeloff Institute of Microbiology, Bulgarian Academy of Sciences, Sofia, Bulgaria; ^2^Laboratory of Clinical Immunology, Department of Clinical Hematology, Sofiamed University Hospital, Sofia, Bulgaria; ^3^Faculty of Biology, Sofia University “St. Kliment Ohridski,” Sofia, Bulgaria; ^4^Neurosurgery Clinic, St. Ivan Rilsky Hospital, Sofia MU, Sofia, Bulgaria; ^5^Department of Tumor Biology, Institute for Cancer Research, Oslo University Hospital, Oslo, Norway; ^6^Department of Molecular Immunology, Institute of Biology and Immunology of Reproduction, Bulgarian Academy of Sciences, Sofia, Bulgaria; ^7^Institute of Biology and Biomedicine, N.I. Lobachevsky University, Nizhny Novgorod, Russia; ^8^Winthrop P. Rockefeller Cancer Research Center, UAMS, Little Rock, AR, United States; ^9^Centre for Bioinformatics, Department of Informatics, University of Oslo, Oslo, Norway

**Keywords:** diagnostic, phage display, antibody repertoire, mimotope, systems immunology

## Abstract

Specific antibody reactivities are routinely used as biomarkers, but the antibody repertoire reactivity (igome) profiles are still neglected. Here, we propose rationally designed peptide arrays as efficient probes for these system level biomarkers. Most IgM antibodies are characterized by few somatic mutations, polyspecificity, and physiological autoreactivity with housekeeping function. Previously, probing this repertoire with a set of immunodominant self-proteins provided a coarse analysis of the respective repertoire profiles. In contrast, here, we describe the generation of a peptide mimotope library that reflects the common IgM repertoire of 10,000 healthy donors. In addition, an appropriately sized subset of this quasi-complete mimotope library was further designed as a potential diagnostic tool. A 7-mer random peptide phage display library was panned on pooled human IgM. Next-generation sequencing of the selected phage yielded 224,087 sequences, which clustered in 790 sequence clusters. A set of 594 mimotopes, representative of the most significant sequence clusters, was shown to probe symmetrically the space of IgM reactivities in patients' sera. This set of mimotopes can be easily scaled including a greater proportion of the mimotope library. The trade-off between the array size and the resolution can be explored while preserving the symmetric sampling of the mimotope sequence and reactivity spaces. BLAST search of the non-redundant protein database with the mimotopes sequences yielded significantly more immunoglobulin J region hits than random peptides, indicating a considerable idiotypic connectivity of the targeted igome. The proof of principle predictors for random diagnoses was represented by profiles of mimotopes. The number of potential reactivity profiles that can be extracted from this library is estimated at more than 10^70^. Thus, a quasi-complete IgM mimotope library and a scalable representative subset thereof are found to address very efficiently the dynamic diversity of the human public IgM repertoire, providing informationally dense and structurally interpretable IgM reactivity profiles.

## Introduction

The repertoire of human IgM contains a considerable proportion of moderately autoreactive antibodies, characterized by low intrinsic affinity/low specificity (1). They function as a first line of defense and as scavengers of senescent cells and debris (2–6) and even in tumor surveillance (7). It is becoming increasingly clear that the human antibody repertoire has an organization similar to that of its murine counterpart (8–12). About one fourth of the murine splenic B lymphocytes that respond to lipopolysaccharide have B-cell receptors that are moderately autoreactive. Practically unaffected by somatic mutations and follicular evolution, the physiological self-reactivities largely overlap with the germline-encoded polyspecific antibodies (13–15). Eighty percent of murine serum IgM falls in this category and is referred to as natural antibodies (nAbs) (16, 17). Apart from the polyspecific splenic B cells, the source of nAbs in mice seems to be mostly a population of B1-related IgM^+^ plasma cells residing in a unique IL-5-dependent bone marrow niche (18).

IgM antibodies appear early in the course of an infection. However, they fall relatively fast, even after restimulation, providing a dynamic signal. By interacting with structures of the self and carrying housekeeping tasks, this part of the antibody repertoire is coupled to changes in the internal environment. Consequently, IgM antibodies have gained interest as biomarkers of physiological or pathological processes (19–23). Yet they remain underused in immunodiagnostics, although their interactions with sets of antigens have been studied in a range of platforms (19, 22–25). The reasons IgM antibodies are rarely considered are probably their low specificity and transitory expression due to which particular specificities are used mostly to detect recent infection.

The study of the IgM repertoire (igome) might be expected to give information about interactions that occur mostly in the blood and the tissues with fenestrated vessels, because, unlike IgG, IgM cannot easily cross the normal vascular wall. Yet IgM tissue deposits are a common finding in diverse inflammatory conditions (26–28) and especially in the disorganized vasculature of the tumors, where they are a key element of the innate immune surveillance mechanism (7, 29, 30). Changes in the IgM repertoire further reflect B-cell function affected by antigenic, danger, and inflammatory signals, but also by anatomical changes leading to vascular permeability or disruption. Thus, IgM repertoire monitoring has the potential to provide clinically relevant information about most of the pathologies involving inflammation and vascular remodeling, as well as all types of cancer.

Our goal was to demonstrate that an essential part of the human polyspecific IgM repertoire involved in homeostasis could be probed by a set of mimotopes, which could be rationally scaled to sizes appropriate for the diagnostic tasks. Essentially, our approach does not specifically target disease specific antibody reactivities but rather the natural antibody repertoire as a universal biosensor of changes of the internal environment. The existing approaches for immunosignature (31, 32) or immunomic (33) analysis of the immunoglobulin repertoires focus mostly on IgG and have used arrays of either 10^2^ proteins or 10^4^-10^5^ random peptides. The IgM repertoire has been previously probed by protein arrays (34), containing a physiologically determined representative set of autoantigens, which is a structurally coarse approach. We set out to explore the feasibility of a method that, similar to the self-protein “homunculus” arrays (15, 23), targets a small set of rationally selected probes but also preserves the structural interpretability of peptides in a format applicable for routine diagnostics.

## Materials and Methods

### Deep Panning

Human IgM was isolated from a sample of IgM enriched IVIg, IgM-Konzentrat (Biotest AG, Dreieich, Germany, generously provided by Prof. Srini Kaveri), whereas human monoclonal IgM paraprotein was isolated from an IgM myeloma patient's serum selected from the biobank at the Center of Excellence for Translational Research in Hematology at the National Hematology Hospital, Sofia (with the kind cooperation of Dr. Lidiya Gurcheva). In both cases, IgM was purified using affinity chromatography with polyclonal anti-μ antibody coupled to agarose (A9935, SIGMA-ALDRICH, USA). A 7-mer random peptide library (E8100S, Ph.D.-7, New England Biolabs, USA) was panned overnight at 4°C on pooled human IgM adsorbed on polystyrene plates at a concentration of 0.1 mg/ml, washed, eluted with glycine buffer at pH 2.7, and immediately brought to pH 7. The eluate was transferred to a plate coated with monoclonal IgM and incubated according to the same protocol, but this time, the phage solution was collected after adsorption and amplified once, following the procedure described by Matochko et al. (35). Briefly, the phage DNA was extracted, and the peptide-coding fragment amplified by PCR. The amplicons were subjected to deep sequencing using the NextSeq platform (Illumina, USA), performed at the Sequencing Core Facility of Oslo University Hospital.

### Patients' Sera

Sera were obtained from randomly selected patients with glioblastoma multiforme (GBM), brain metastases of breast (MB) or lung (ML) cancers, and non-tumor-bearing patients (C) (herniated disc surgery, trauma, etc.) of the Neurosurgery Clinic of St. Ivan Rilski University Hospital, Sofia. The samples were acquired according to the rules of the ethics committee of the Medical University in Sofia, after its approval and obtaining informed consent. The sera were aliquoted and stored at −20°C. Before staining, the sera were thawed; incubated for 30 min at 37°C for dissolution of IgM complexes; diluted 1:100 with phosphate-buffered saline (PBS), pH 7.4, and 0.05% Tween 20 with 0.1% bovine serum albumin (BSA); further incubated for 30 min at 37°C; and filtered through 0.22-μm filters before use. The serum IgM reactivity was analyzed on different sets of peptides defined in microarray format.

### Peptide Microarray

The customized microarray chips were produced by PEPperPRINT™ (Heidelberg, Germany) by synthesis *in situ* as 7-mer peptides attached to the surface through their C-terminus and a common spacer GGGS. The layout was in a format of a single field of up to 5,500 or five fields of up to 600 peptides in randomly positioned duplicates. The chips were blocked for 60 min using PBS, pH 7.4, and 0.05% Tween 20 with 1% BSA on a rocker; washed 3 × 1 min with PBS, pH 7.4, and 0.05% Tween 20; and incubated with sera in dilutions equivalent to 0.01 mg/ml IgM (~1:100 serum dilution) on a rocker overnight at 4°C. After 3 × 1-min washing, the chips were incubated with secondary antibodies at room temperature (RT), washed, rinsed with distilled water, and dried by spinning in a vertical position in empty 50-ml test tubes at 100 × g for 2 min.

### Microarray Data Analysis

The microarray images were acquired using a GenePix 4000 Microarray Scanner (Molecular Devices, USA). The densitometry was done using the GenePix® Pro v6.0 software. All further analysis was performed using publicly available packages of the R statistical environment for Windows (v3.4.1) (Bioconductor; Biostrings, limma, pepStat, sva, e1071, Rtsne, clvalid, entropy, RankProd, multcomp, etc.) as well as in-house developed R scripts (https://github.com/ansts/IgMimoPap1 and https://github.com/ansts/IgMimoPap2). For algorithm details, see Supplementary Methods.

### BLAST Search for Homologous Peptides

Sections of protein sequences homologous to the studied peptides were identified using the blastp function and the non-redundant human protein database of NCBI (36–38). The parameters were automatically adjusted for short sequences, and the results further restricted to those with a minimum of six positive positions and a minimum of six identity position with no gaps. The alignments are provided as Supplemental Files. This search was performed for three of the libraries: SYM, RND, and NGR. The NGR sequences introduced as negative control in the library SYM were removed so it represented only 519 sequences. For all libraries, the hits of each peptide were classified into (1) immunoglobulin if at least one of the hits was in the heavy or light chain of the immunoglobulin genes, (2) non-immunoglobulin (hit in any other human protein sequence), and (3) no hit. The number of hits in immunoglobulin J region sequences greatly exceeded the length of those in other parts of the immunoglobulin sequences, and also J regions are naturally overrepresented in the database. Therefore, we considered with some approximation that the majority of the immunoglobulin hits were in J regions and if occasionally not, then in variable regions. The proportion of hits in immunoglobulin sequences were compared using the chi-square test.

## Results

### Selection of 7-Mer Mimotopes

**Figure 1:**
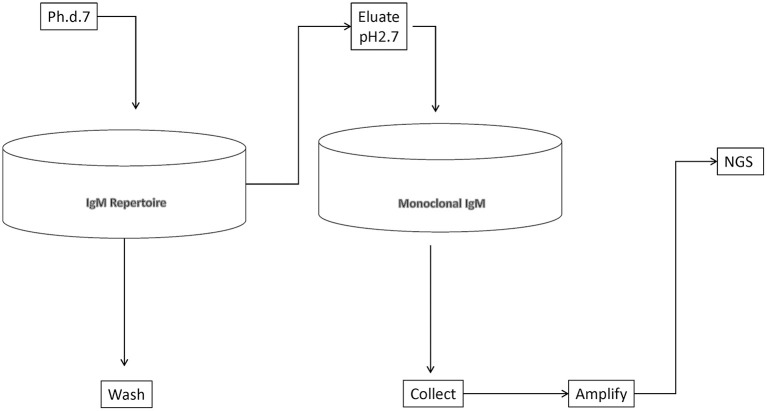
Schematic representation of the deep panning experiment.

We set out to define as complete as possible a library of mimotopes of the normal human broadly expressed IgM repertoire. To this end, we chose to pan a commercially available 7-mer random peptide phage display library (Ph.D.-7, New England Biolabs) of diversity 10^9^. Thus, the size of the mimotopes would be in the range of the shortest linear B-cell epitopes in the IEDB database (http://www.iedb.org/). At the same time, an almost complete diversity of sequences of that length could be interrogated. As a repertoire template, we used an experimental preparation of human immunoglobulins for intravenous use enriched in IgM. It represents a pool of the repertoire of ~10,000 healthy donors. Total IgM was isolated from it by affinity purification. The phage eluted from the IgM repertoire was adsorbed on a monoclonal IgM to filter out phage binding to the constant regions, thereby focusing only on the mimotopes (Figure 1). The peptide inserts were amplified and deep sequenced using the approach described by Matochko et al. (35). Two separate experiments starting with 20% of the original phage library were performed (experiments A and B), whereas in a third one (C), the starting point was a preamplified 20% sample of the original library. The yield was 688,860 (experiment A), 518,533 (experiment B), and 131,475 (experiment C) unique reads. Based on the distribution of the reads by copy number in the selections from the native and preamplified library, two thresholds were determined, that is, 2 and 11 copies, and the reads within these limits (exclusively) were considered further (see Supplementary Methods). The lower limit ensured the acceptable sequencing error level, and the upper was used to avoid overgrowing phage clones.

### Sequence Properties of the Mimotope Clusters

**Figure 2:**
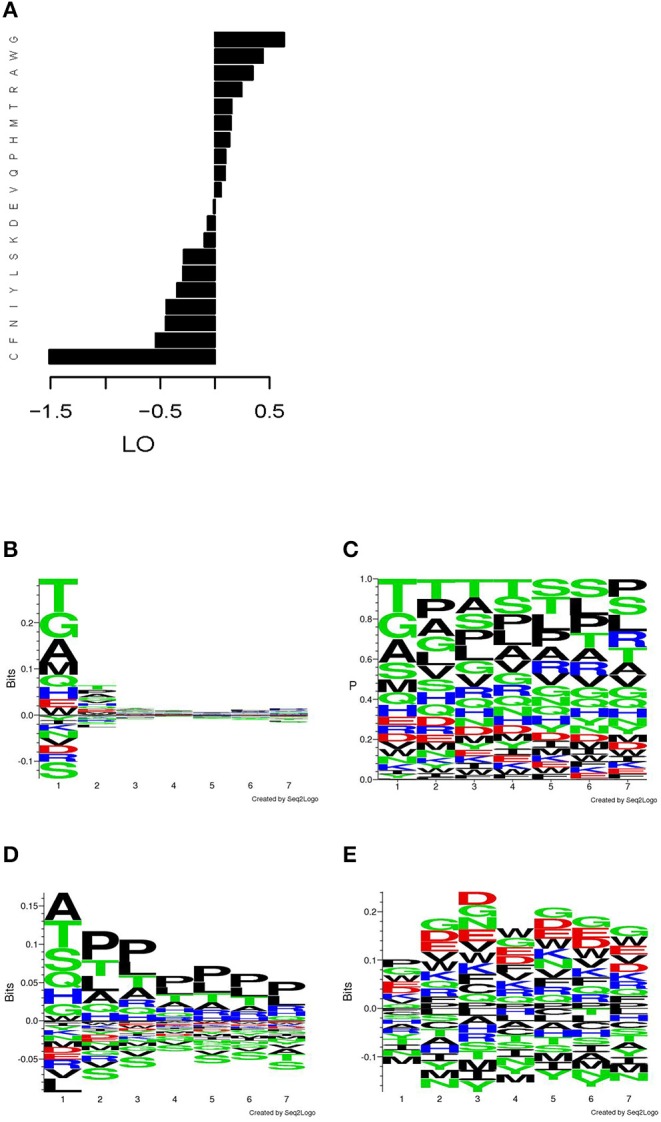
Distribution of the amino acid residues in the mimotope library. **(A)** Log odds (LO) relative to background frequencies. **(B)** Sequence logo of the LO by position relative to the overall background frequencies. **(C)** Sequence logo of the frequencies by position. **(D)** Sequence logo of the LO by position in an amplified Ph.D.-7 phage library without ligand selection relative to the overall background frequencies (based on W. L. Matochko, R. Derda. “Error analysis of deep sequencing of phage libraries: peptides censored in sequencing”. *Comput Math Methods Med*, 2013, 491612. 2013., http://www.chem.ualberta.ca/~derda/parasitepaper/rawfiles/PhD7-GAC-30FuR.txt). **(E)** Sequence logo of the LO by position relative to the frequencies by position in the amplified, unselected library shown in **(D)**. The skewing of the distribution in the free N-terminus appears to be property of the library, whereas the selection by the IgM repertoire leads to slight skewing in the middle of the sequence toward negatively charged residues, glycine and tryptophan.

The overall amino acids residues frequencies (AAF) in the mimotopes selected from the phage library showed a skewing in favor of G, W, A, R, T, H, M, P, and Q and against C, F, N, Y, I, L, and S (Figure 2A), when compared with the average overall amino acid frequencies of the Ph.D.-7 library. When studied by position, the distribution of AAF visualized by the respective sequence logos showed a highly skewed distribution for the N-terminus (Figure 2B). The actual frequencies by position are shown in Figure 2C. The residues of W, D, and E appear in similar frequencies, but owing to the much lower abundance of W in the phage library, in Figure 2A, it comes up as selected and D and E as slightly disfavored. The N-terminal frequency skewing and the preference for A, P, and T proved to be properties of the library. This became evident after comparing the AAF by position of a non-selected but amplified library [based on the data from Matochko et al. (35) Figure 2D]. The evidence of selection by IgM stood out in the distribution by position only after using the position weight matrix (PWM) of the non-selected amplified library as background frequencies to describe the actual enrichment in our mimotope library (Figure 2E). It showed a slight divergence from the background distribution of the frequencies in the middle of the sequence. Overrepresentation of proline in positions 2–7 appears to be a property of the amplified library, whereas the IgM binding selected for negatively charged residues, as well as glycine and tryptophan.

**Figure 3:**
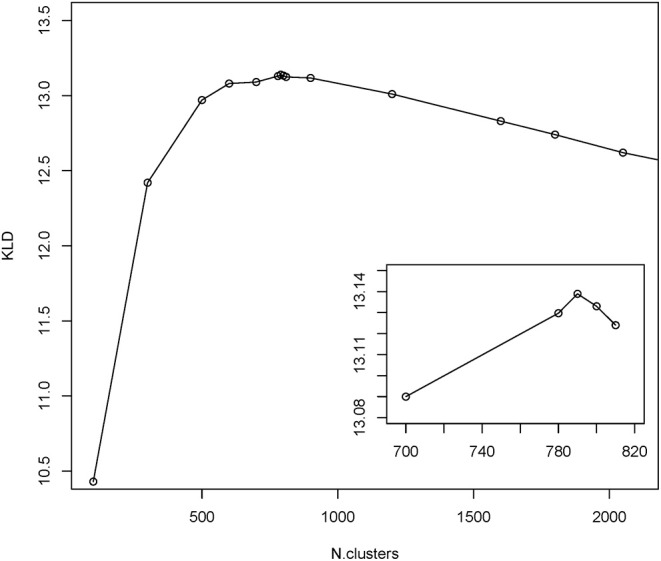
Results from GibbsCluster of the mimotopes. Different predefined numbers of clusters were screened for the quality of clustering as measured by Kullback–Leibler divergence (KLD). The inset shows the amplified part around the peak KLD values.

To gain insight into the mimotope sequence space, the set of 224,087 selected mimotope sequences was subjected to clustering using the GibbsCluster-2.0 method (39). This algorithm was applied originally for inferring the specificity of multiple peptide ligands tested on multiple major histocompatibility complex (MHC) receptors. The number of clusters was optimized by scanning the range of 100 to 2,500 clusters and using the Kullback–Leibler divergence (KLD) criterion. This is an information theory-based measure of similarity between two distributions. Here, it was used to compare the sequence alignments by cluster to the background model of random sequences (39). The cluster number scan indicated optimal clustering in 790 clusters (Figure 3). The range of cluster numbers was chosen on the basis of biological relevance (100) and computational complexity (2,500), and the choice proved suitable as much as there was a single global maximum of the criterion in this range. Position-weighted matrices were calculated next from each cluster (Supplement File 2).

### Generation of a Mimotope Library for Practical Probing of the IgM Igome

The mimotope library of more than 200,000 sequences is a rich source of potential mimotope candidates for vaccine or diagnostics. Yet the size of a probe containing an array of 10^5^ peptides is impractical for routine diagnostic use. A way to scale down the mimotope probe array would be to include a representative sequence of each of the naturally existing 790 clusters. Only the sequence with the top score from each cluster was kept as a mimotope prototype for the cluster. These are expected to sample evenly (symmetrically) the mimotope sequence space, as ensured by the GibbsCluster algorithm.

The mimotope sequence diversity in each cluster was significant. Thus, cutting down so much the list of representative sequences would seem counterintuitive with respect to our goals. Nevertheless, this approach was chosen because such a symmetric sequence set was hypothesized to address a much wider range of IgM clones than the mere number of mimotopes chosen because of the well-documented polyspecificity of the majority of the antibodies probed.

The sequence clusters were found to vary with respect to the probability of random occurrence of such a group of sequences, which was used to rank them by significance (Supplementary Methods). As the optimal format of the microarrays used included five fields of 600 peptides, the top 519 clusters cluster representative sequences were considered further. Finally, 75 sequences from the negative control library NGR (see below) were added to a total of 594 peptides. This library was labeled SYM for symmetric.

The relevance of this mimotope library to the complete repertoire of broadly expressed IgM reactivities and the scope of their diversity could be established by comparing several different peptide libraries with different properties.

An alternative sequence library was constructed *in silico* to check the representativeness of the selected mimotope set and the relevance of the clustering found. To this end, 2.3 × 10^6^ random 7-mer sequences were scored and ranked according to their similarity to each of the 790 clusters of mimotopes defined above. The random sequences that were the least related to any of the clusters in the selected library were used as a negative control (library NGR—see Supplementary Methods).

Other libraries of peptides generated for further comparison were as follows: (1) uncertainly clustered sequences as reflected in their KLD scores as shown by the GibbsCluster algorithm (NG1 and NG2); (2) two groups of five high scoring clusters as lower diversity libraries (C5_1 and C5_2); (3) random 7-mer sequences predicted to belong to some of the five highest scoring clusters based on PWM profile scores (C5P); and (4) random 7-mer sequences (RND) (see Table 1 for description of all libraries).

**Table 1:** Libraries of 7-mer peptides studied.

**Library**	**Description**	**Number of peptides**
SYM	A library that samples symmetrically the mimotope sequence space. Contains the sequence with the highest score for the respective position weight matrix from each significant cluster (significant clusters are those for which the number of sequences with more than median PWM score is greater than the expected number of occurrences of such score in random peptides—*p* < 0.0001 by binomial test).	594
C5_1	A group of 5 of the 288 clusters with best binomial *p* < 1e−16: clusters #2, 6, 9, 10, and 11. This library is an example of a lower diversity set.	600
C5_2	A group of 5 of the 288 clusters with best binomial *p* < 1e−16: clusters #115, 61, 55, 53, and 258. This library is an example of lower diversity set.	1,193
C5P	150 random^*^ sequences with log odds scores greater than the median score of the respective cluster for each of 5 clusters (clusters #2, 6, 9, 10, and 11). This library tests the capacity of the sequence profiles to capture the antigenic properties of the mimotopes.	750
NG1	The lowest scoring sequence (using KLD) from each significant cluster. These sequences are least certain to belong to any of the 790 clusters.	594
NG2	Among the set of the lowest scoring sequences (NG1) using GibbsCluster's own “Corrected” score—those with score <5 (39). Another version of the previous library.	82
NGR	The max scores for each of a set of 2 × 10^6^ random^*^ 7-mer sequences after testing against each cluster PWM are ranked, and the sequences with the lowest ranks are retained representing sequences least related to the mimotope library.	753
RND	800 random peptides.	800
	Total	5,366

*The number of sequences per library was constrained by the size of the microarray chip used for their testing. PWM, position weight matrix*.

* *The random sets are constructed with underlying frequencies in phage display library Ph.D.-7*.

### Comparison Between Libraries

**Figure 4:**
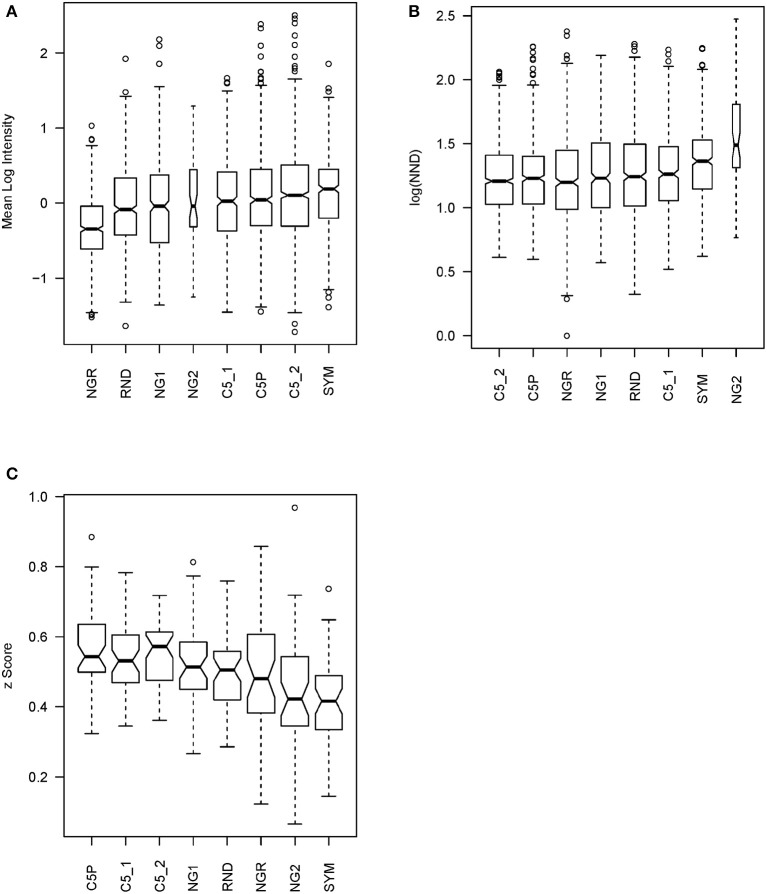
Statistics testing the libraries' capacity to probe the mimotope reactivity space. **(A)** Mean reactivity of each peptide across patients grouped by library. The optimized library SYM has the highest reactivity. For library content, see Table 1. **(B)** Mean nearest neighbor distance of the peptide profiles across 10 patients in each library. **(C)** Mean correlation of patient profiles across the peptides in each library compared after *z* transformation. This test has a similar meaning as the previous but is performed on the transposed matrix. The optimized library SYM provides the least correlated patient reactivity profiles, which indicates a high potential to reflect the natural diversity of the human population but increases the requirements for the size of the teaching sets to extract models of good generalization. The optimized library SYM appears to sample the mimotope reactivity space evenly. The width of the bars is proportional to the size of the sample.

IgM reactivity in sera from patients with GBM (*n* = 3), MB (*n* = 3), and non-tumor-bearing neurosurgery patients (C, *n* = 4) was analyzed using the sets of peptides described in Table 1. The peptide libraries were synthesized in an oriented (C-terminus attached) planar microarray format. In the first round of experiments, the eight different libraries defined were compared based on the IgM reactivity in the sera from 10 patients (Supplementary Figures 4, 5). The data on the mean serum IgM reactivity of the peptides with the different sera, grouped by library, were used to compare the libraries for their overall reactivity using linear models (Figure 4A). The proposed optimized small library (SYM) had significantly higher (*p* < 0.001) average reactivity than NG1, RND, or NGR. Interestingly, the library NGR, which was *in silico* purged of sequences scoring high with the 790 profiles of mimotope clusters defined, had indeed the lowest reactivity. It was significantly lower than that of both the weakly clustering peptides (NG1) and the random sequences (RND) (Supplementary Table 1). This is considered an indication that the mimotope library of the order of 10^5^ sequences is a quasi-complete igome image. Also, this fact is in support of the hypothesis that the 790 cluster profiles summarize in sufficient detail the salient sequence features that define the mimotopes of the public human IgM repertoire.

Next, the capacity of the different libraries to sample symmetrically the space of 7-mer IgM mimotope reactivities in the IgM repertoire was tested. The mean nearest neighbor distance (MNND) was used for that purpose as a statistic indicating clustering of the data. Peptides that have similar reactivity profiles with different sera (thus carrying redundant information) would map to points in the reactivity space that lie close to each other. This clustering in some regions of the space would lead to a lower MNND. The library SYM ranked second only to NG2 (Figure 4B) by this parameter and had a significantly higher MNND than all the other libraries (Supplementary Table 2).

The correlations between the patient profiles of reactivity were also used as a measure of the capacity of the libraries to extract information from the IgM repertoire. We tested all pairwise correlations between the patient profiles with the peptides from a given library. After *z* transformation of the correlation coefficients to allow for comparison by linear models, the means of those *z* values for each library were used to compare the libraries (Figure 4C). Again, the SYM library exhibited the lowest mean correlation—significantly lower than the correlation between the reactivities with the other libraries except for NGR and NG2 (Supplementary Table 3).

Finally, all three criteria were summarized using a rank product test, which proved that reactivity with SYM stands out from all the other tested libraries as the best among them for probing the IgM repertoire (Table 2).

**Table 2:** Rank product test of three criteria for optimal mimotope library.

**Library**	**Rank products**	***p* value**
C5_1	4.380	0.650
C5P	5.518	0.853
NG1	5.313	0.823
NG2	2.154	0.099
NGR	5.241	0.812
C5_2	4.579	0.692
SYM	1.260	0.007
RND	4.820	0.739

### Visualization of the Mimotope Sequence Space

**Figure 5:**
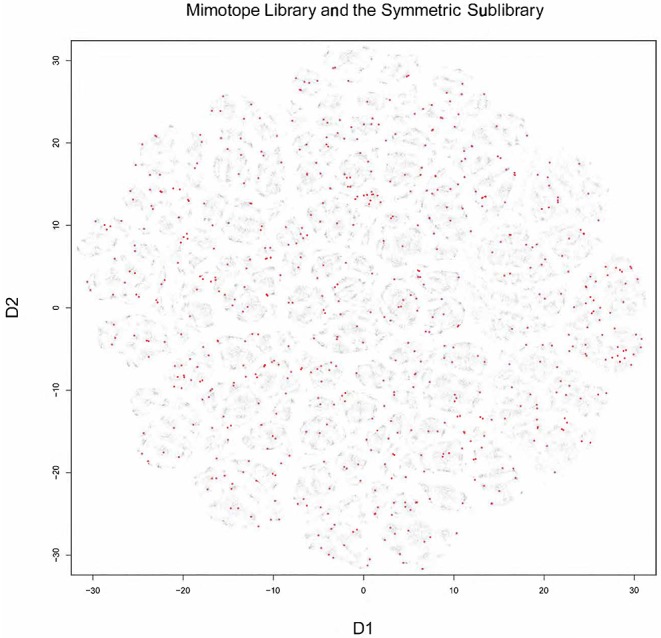
Visualization of the 7-mer mimotope sequence space with the optimized library SYM marked in red. Although individual GibbsCluster defined clusters do not coincide with those shown by t-sne, the mapping of the optimized library apparently probes quite uniformly the mimotope sequence space. t-sne, T-distributed stochastic neighbor embedding.

T-distributed stochastic neighbor embedding (t-sne) was used to visualize the structure of the mimotope sequence space as represented by the general mimotope library produced by deep panning. To represent the sequences as vectors of real numbers, each amino acid residue was represented by five scores based on the z1–z5 scales published by Sandberg et al. (40) (see Supplementary Methods for details). Thus, each 7-mer sequence was parameterized as a 35-dimensional vector. These vectors were then represented in two dimensions by t-sne transformation. The map of the mimotope library, thus generated, resembled that of an equal number of random 7-mer sequences constructed using the residue background frequencies of the phage display library (Supplementary Figure 7). Next, the representation of some of the clusters of mimotopes described above was mapped in this new mapping. Although the five most significant among 790 sequence clusters (the C5_1 library, Supplementary Figure 7) mapped to rather scattered clusters in the t-sne representation, the mapping of the optimized library (SYM) still covered symmetrically the mimotope sequence space (Figure 5). Both the clustering and the mapping do not give unique solutions and fail to capture the full information in the general mimotope data set. Yet the symmetry of the optimized library SYM, designed rationally on the basis of the clustering, is nevertheless reflected in the t-sne mapping.

### SYM Overrepresents Mimotopes of Idiotopes

An important aspect of the usage of mimotopes as igome probes is their interpretability. Using large mimotope libraries provides an opportunity to generalize the type of structures targeted by the antibody repertoire studied. The numerous tests may allow for signal to emerge despite the noise due to poor representativeness of conformational epitopes, polyspecificity, mimotope/epitope sequence length disparity, etc.

**Figure 6:**
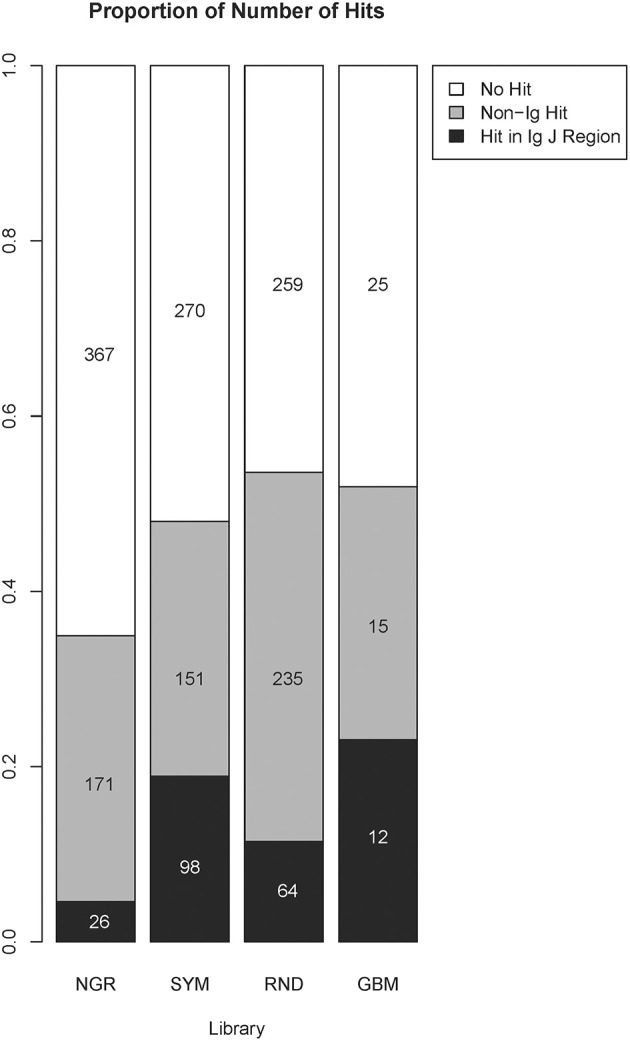
Comparison between the number of homologous sequences found by BLAST search of the non-redundant human protein database classified as immunoglobulin J regions or non-immunoglobulin hits. The numbers and the size of the shaded bars correspond to the number of peptides having this type of homolog in the targeted database, and the proportion these peptides represent the volume of the library. A peptide was considered to be homologous to an immunoglobulin J region if the search returned at least one hit in a variable J region. The parameters were automatically adjusted for short sequences, and the results further restricted to those with a minimum of six positive positions and a minimum of six identity position. The alignment length was set equal to the number of positive positions, and no gaps were allowed. The proportions were compared using the chi-square test followed by pairwise multicomparison with false discovery rate correction (overall, *p* = 2 × 10^−16^; SYM/Ig vs. RND/Ig, *p* = 1.2 × 10^−6^; SYM/non-Ig vs. RND/non-Ig, *p* = 3.6 × 10^−9^; NGR/Ig vs. RND/Ig, *p* = 2 × 10^−8^; GBM/Ig vs. RND/Ig, *p* = 0.045). GBM, glioblastoma multiforme.

To explore the capacity of the library SYM to reveal general properties of the antigens targeted by the natural IgM repertoire, we used NCBI blastp program to find SYM homologous short sequences in the non-redundant (nr) database of human proteins (36, 37). The parameters of the search were automatically adjusted to short sequences. Owing to the extremely short length of the query sequences, the expectation values were high. The proteins containing sequences homologous to SYM mimotopes were very often immunoglobulin junction region. This is not surprising as the diversity of the immunoglobulin J regions included in the human nr database is on the same scale as the overall diversity of the proteome. We were somewhat surprised, though, to see a highly significant preference to immunoglobulin J regions in the alignments of the SYM library as compared with the RND and especially with the NGR library as well as in the RND compared with NGR (Figure 6). The NGR sequences attached to SYM as controls were excluded for this search, yielding only 519 mimotopes. Despite the small number of sequences, the profile derived for GBM (see below) had even higher number of homologies in J regions.

### Diagnostic Potential of a Rationally Designed Restricted Mimotope Library

To test the diagnostic potential of the SYM library, we chose to look for reactivity profiles able to separate sera from patients with different brain tumors. Although somewhat questionable, our expectation to find IgM repertoire correlates of brain tumor diseases was justified by (1) reports by Merbl et al. (23) that both IgG and IgM natural autoreactive antibody profiling with self-antigens can discriminate between murine tumors and from O'Donnell et al. (41) that 3 × 10^5^ random peptide-based profiling of antibody repertoires can discriminate GBM from other tumors; (2) reports indicating the role of natural IgM antibodies in the immune surveillance against tumors (7, 30–39, 41–43); and (3) reports of diagnostic or therapeutic natural IgM binding-defined tumor antigens (44–46). Furthermore, cancer is a suitable test for the natural IgM igome's diagnostic utility because of the localized inflammation and shedding of tumor-specific and tumor-associated antigens. Therefore, this type of pathology serves better to test a “biosensor” property of the functional repertoire. It would surely be easier to show SYM profile changes studying diseases affecting the repertoire itself, which cause large-scale distortion like hyper-IgM syndrome, myeloma, and reconstitution after ablative therapy, but this was deemed insufficient.

For this assay, we used sera from a set of 34 patients with brain tumors. The main goal was a “proof of principle” test demonstrating the capacity of the assay to provide mimotope reactivity profiles suitable for building predictors for randomly selected pathology. The distribution of patients by diagnosis (GBM, ML, MB, and C) is shown in Table 3. The microarray data were cleaned and normalized locally and globally, and the group sizes were balanced, which warranted the use of the ComBat function (47) for the batch effect compensation. The reactivity data, thus obtained, represented 28 patients' serum IgM binding to 586 peptides. No reactivity was found specifically expressed in any of the diagnostic groups. Each patient's serum, though, reacted significantly with most of the mimotopes as compared with the pool of the rest of the patients. The reactivities with individual sera were between 339 and 390 (mean, 368) out of 586. Altogether, 582/586 reactivities were significant in at least one patient.

**Table 3:** Patients tested using the optimized library.

		**Batches**			
**Diagnosis**	**Abbr**.	***G***	***P***	***R***	**Total**
Non-tumor bearing (control)	C	1	3	4	8
Glioblastoma multiforme	GBM	2	4	9 (5)^*^	15 (11)^*^
Lung cancer (brain metastasis)	ML	2	4	3	9
Breast cancer (brain metastasis)	MB	0	0	2 (0)^*^	2 (0)^*^
	Total				34

* *To balance the group sizes between batches, only 5/9 GBM samples from batch “R” were used, and the breast cancer cases were omitted before batch compensation using the ComBat function. All cases in batch “R” were used in the validation step; those omitted from the training step served as a testing set*.

A two-dimensional projection of the cases on the 582 positive reactivities by multidimensional scaling (MDS) showed no separation (data not shown). This is expected because the peptide library is not targeted to any particular pathology. It represents rather a universal tool for IgM repertoire probing and mapping to a highly multidimensional feature space. The information in the reactivity profiles when all features are used is so rich that it makes practically each patient unique and a generalization impossible. In addition, the “curse of dimensionality” makes differentiating in 582 dimensional space hard. Therefore, a feature selection step would be necessary to construct a predictor for any diagnostic task.

**Figure 7:**
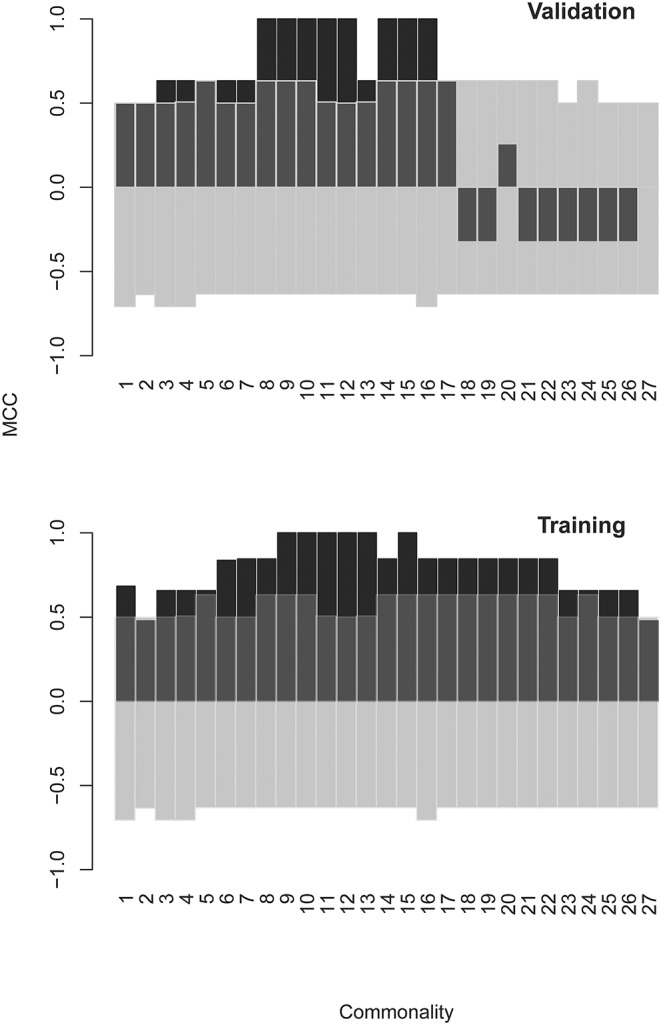
Matthews correlation coefficient (MCC) as a measure of the quality of SVM models using different optimized feature sets. The models were constructed using GBM specific feature sets derived by a combination of filtering and backward and forward feature selection steps. Finally, consensus feature sets were formed from at least *n* “leave one out” groups of patients: *n* = 1 means all features from all groups were pooled, and *n* = 27 means the set contains only the three features found in all 27 groups (there was only one feature found in all 28 groups). The testing set consists of the cases in batch “R” that were omitted from the batch compensated pooled patient group. The model predicts correctly the classification of these cases. As the values in batch “R” were not subject to batch compensation, the validation also serves as a control against artifacts introduced by the ComBat function. The transparent bars indicate the 5% and the 95% confidence limits of MCC calculated on the basis of 1,000 scrambled matrices. SVM, support vector machine; GBM, glioblastoma multiforme.

A combination of filtering and wrapping feature selection techniques was applied next. The filtering method used was a selection of individual features with highly significant expression in at least one patient. The wrapping techniques were recursive feature elimination followed by a forward selection algorithm. The feature to remove (respectively to add) at each step was selected so as to improve maximally the separation of the patient data clusters of interest when mapped on the remaining features. This iteration was repeated until no further improvement of the separation is possible (see Supplementary Methods section for details). This algorithm produced SYM subsets of 60 up to 220 features depending on variations of the clustering criterion (recursive feature elimination derived sets [RFEDSs]). Using this approach, we tested the capacity of the respective RFEDSs to separate dichotomously GBM from the rest of the cases. For the consecutive steps, the cases omitted before the batch compensation were used as a testing set. A support vector machine (SVM) model based on GBM-RFEDSs separated well the patient groups in the training set. Still, it suffered from overfitting when tested in a leave-one-out validation (data not shown) performed within the limits of the overall training set. To achieve generalization, next, we explored the variation of the GBM-RFEDSs using patient data sets that differed by 2/28 cases (using the leave-one-out scheme—Supplementary Methods). It was surprising to find that so similar patient groups produced different GBM-RFEDSs that contained between 15 and 194/582 reactivities (median = 54) with only one feature common for all GBM-RFEDSs generated. The reason for this could be the variability between individuals and the capacity of the mimotope library to reflect it. Another reason could be the hypothesized highly convoluted nature of the profiles—each IgM clone should be represented by a number of reactivities, and each peptide possibly reacts with more than one clone. Nevertheless, a good prediction both of the training and of the testing sets using SVM was possible when combining features that recur widely in the GBM-RFEDSs. The best performing feature set represented a pool of 43 features each found in at least 15 of the 28 GBM-RFEDSs (Figure 7, see Supplementary Methods for further details).

**Figure 8:**
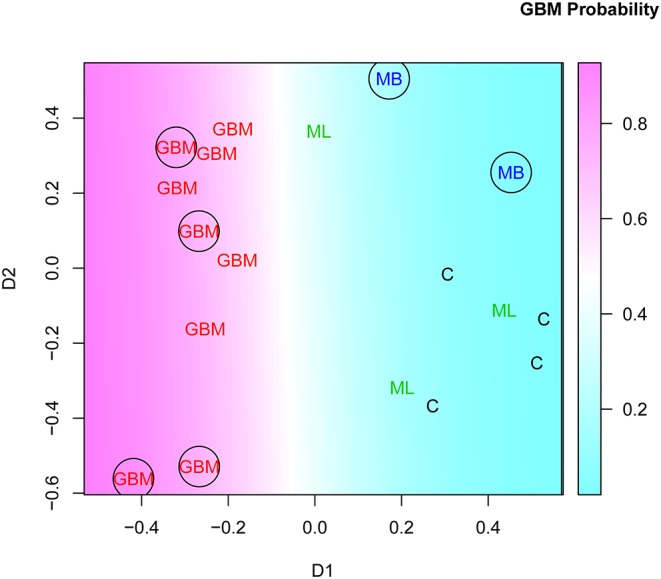
Multidimensional scaling plot of cases in batch “R” based on the set of GBM-related features found in at least 15/28 of the “leave on out” patient groups. See Figure 7 and Supplementary Methods for details. The encircled points correspond to the testing set. GBM, glioblastoma multiforme.

Interestingly, this two-stage feature selection strategy (bootstrapping RFEDS variability and pooling recurring features) helped improve the generalization considerably. Testing of the model of 43 dimensional data with just a few cases is impractical. Therefore, the dimensionality of the IgM reactivity data was reduced from 43 to 2 using MDS. The SVM model, constructed on the basis of the two surrogate features obtained by MDS, successfully classified the GBM and non-GBM cases not only of the training but also of the testing set of sera (Figure 8).

Thus, we were able to show that a rationally designed small library of 586 IgM mimotopes contains potentially a huge number of mimotope profiles that can differentiate randomly selected diagnoses after appropriate feature selection.

## Discussion

High-throughput omics screening methods have extracted profiles from different dynamic diversities (proteome, genome, glycome, secretome, etc.) and used them as biomarkers. The use of the antibody repertoire as a source of biomarkers has also been defined and approached in multiple ways. First came the technically minimalistic, but conceptually loaded, semiquantitative immunoblotting, developed 20 years ago (48–53). The further development produced methods that have been referred to as functional immunomics (33) in terms of protein reactivities or as immunosignaturing (31) in terms of random peptide libraries or described as a deep panning technique (54) in terms of complete set of mimotopes (igome) selected from random phage display libraries. Here, we describe the design of the first mimotope library for the analysis of the human IgM repertoire of reactivities recurrent in most individuals also referred to as public repertoire (12, 55, 56). The focusing on broad reactivities is a consequence of panning on IgM pooled from at least 10,000 donors, which dilutes the unique specificities.

The deep panning approach relies on next-generation sequencing (NGS) and thus requires balancing between sequence fidelity and diversity. Even with diversity affected by discarding sequences of one and two copies on the one hand, and overgrowth of phage clones on the other, our strategy still manages to find a general representation of the mimotope sequence space by identifying clusters of mimotopes. This relatively small set of sequence classes is hypothesized to be related to the modular organization of the repertoire defined previously (57). Alternatively, it can also be an artifact of the sequence clustering algorithm depending on the size and diversity of the sequence set. In the latter case, the clustering may not reflect a biological phenomenon but still provides a tool for the classification of the sequences.

The central role of prolines in the nAb mimotopes has been observed previously (58). Tchernychev et al. also used a phage display library. Now, it is clear that the high proline content of nAb mimotopes that they found is related to the bias of the particular phage display library. This property of the library may facilitate the discovery of mimotopes because prolines are associated with turns and flanking structures and proline abundance also reduces the entropic component of the binding. In our experiments, the selection by the IgM repertoire led to an enrichment of tryptophan and negatively charged residues in the middle of the sequences. This suggests that the broad IgM reactivity has a preference for loop-like mimotopes (facilitated by the presence of prolines) with negative charges. The abundance of tryptophan is also interesting in terms of its propensity (together with proline) to mimic carbohydrate structures (59).

The mimotope library of diversity 10^5^ derived by deep panning reflects the recurrent IgM specificities found in the human population. A library of random peptides with sequences selected to be least related to the observed 790 cluster profiles reacted very weakly with IgM from patients' sera. This fact suggests that not only does the library of a little over 200,000 mimotopes represents the IgM mimotope space but also that the 790 cluster profile matrices are collectively a promising model of it. The good coverage of the IgM reactivity space by this mimotope library most probably is facilitated by the polyspecific binding of IgM and the small, flexible peptides.

Although the large mimotope library can be used as is in peptide arrays when applicable, its size is not very practical for routine diagnostics. The classification in 790 clusters was used to produce a smaller and more applicable library, SYM, for clinical use. It contains basically representative sequences from the most significant clusters. SYM represents more efficiently the mimotopes' main reactivity patterns found in the phage selection experiment when compared with seven other libraries chosen to represent key alternative concepts. The precision of that representation can be adjusted by expanding the small library if necessary. Including more mimotopes from the set of 224,087 can be done in a similar fashion, sampling further the existing sequence clusters. Another improvement may be to include a couple of related sequences to each of the mimotopes, for example, those immediately adjacent in the same cluster, for a statistically robust signal.

An interesting though not unexpected property of the public IgM igome found is its idiotypic connectivity. Overlap with immunoglobulin variable domain J regions proved the prominent feature of the human protein sequence fragments homologous to the peptides in SYM library. The actual epitopes of IgM should be mostly conformational. Nevertheless, both linear idiotypic epitopes (idiotopes) and fragments of them are probably represented in the CDR3 loops so as to produce statistically detectable signal in the BLAST results. It has long been known that linear epitope models yield clear structural idiotypic representation in CDR3 loops (60). Interestingly, the overall number of hits in the human proteome for the SYM library was smaller than that for the random library RND, which may reflect the overall tolerance of the igome for self to the background of higher idiotypic connectivity. The considerable self-referential (immunoglobulin-immunoglobulin) component of the igome means that the signal that can be read off it as a biosensor should be viewed in terms of global connectivity perturbations at least as much as in terms of local antibody–autoantigen interactions. Indeed, this idiotypic component is preserved and even somewhat enhanced in the specific profile found for GBM.

SYM could be used as a tool for the study of the IgM repertoire, as a source of mimotopes for design of immunotherapeutics (61–64), but mostly it may be applied as a multipurpose diagnostic tool. As a diagnostic tool, SYM has some key properties that distinguish it from other omics sets. When used to probe sera from patients with different brain tumors, no single reactivity correlated strongly with a whole diagnostic group. Still, quantitative profiles of subsets of reactivities collectively could separate the diagnoses by decision boundaries, which can be non-linear. Thus, appropriate feature selection algorithms are essential for the design of predictors based on the natural, polyspecific igome. With the use of the proposed algorithms, the typical feature sets tuned for dichotomous separation of diagnoses contained between 28 and 111 sequences (median = 66). Keeping only features recurring in at least half of the sets generated in the bootstrapped feature selection algorithm helped remedy the overfitting of the models and achieve the necessary generalization.

The optimal feature set for GBM diagnosis we find has 43 mimotopes. If the library provides in the order of 500 significant reactivities and the profiles are typically of around 50 features, the theoretical capacity of this approach is >10^70^ different subsets. This is an estimate of only the qualitative outcome—presence or absence of reactivity. Thus, the information provided by a typical IgM binding assay with the library is probably enough to describe any physiological or pathological state of clinical relevance reflected in the IgM repertoire. Of course, this is just an estimate of the resolution of the method. The number of naturally occurring profiles and their correlation with clinically relevant states will determine the actual capacity. Another important consideration is the significant probability to find profiles correlating to any state by chance. Therefore, extensive testing of the models to prove their ability to generalize is indispensable.

The novelty of our approach is based on the combination of several previously existing concepts.

First, early studies have argued that the physiologically autoreactive nAbs comprise a consistent, organized immunological compartment (50, 53, 65–68). The consistency of the nAb self-reactivity among individuals was considered evidence for the existence of preferred self-antigens. Such “public reactivities” are most probably related to the germline repertoire of antibodies generated by evolutionarily encoded paratope features and negative/positive selection (34, 69, 70). They were hypothesized to represent the repertoire compartment characterized also by idiotypic interactions (71). These antibodies were targeted using protein microarrays, the utility of which has been previously demonstrated (23, 33, 34, 57). Recently, the existence of structurally distinct public V-regions has been analyzed using repertoire sequencing (12), noting again that they are often found in nAbs. If the repertoire should be read as a source consistent patterns that can be mapped to a wide variety of physiological and pathological states, the public natural IgM autoreactivity seems to be a suitable but underappreciated compartment.

Second, germline variable regions are characterized by polyspecificity or cross-reactivity with protein and non-protein antigens (14). It seems that going for epitopes could be a way to approach the repertoire convolution. Yet the actual epitopes will be mostly conformational and hard to study. In similar tasks, mimotopes are often used (72–75). On the other hand, M. H. Van Regenmortel argues that mimotopes are of little use to structural prediction of a B-cell epitope (74). Apparently, this is less of a problem when the whole repertoire is used to address classes of epitopes whereby a statistically significant signal can be detected.

Third, peptide arrays have been used for some time now as probes of the antibody repertoire (54, 76–79). This includes the use of random peptide arrays for extracting repertoire immunosignatures (32) or deep panning of phage display libraries to analyze antibody response (54). Here, we combined these two approaches. An antibody can often cross-react with a linear sequence that is part of the nominal conformational epitope (74). The 7 aa residue long peptides can be viewed as a simplified set of long “syllables” in the epitope “vocabulary” that can cross-react with the respective antibodies. They represent also a set of small complete epitopes. For instance, in the Immunoepitope Database (http://www.iedb.org) collection of 4 × 10^4^ linear B-cell epitopes close to 5,000 entries are <8 residues long. Thus, the peptide length of seven residues makes possible the interrogation of the repertoire with a library of 10^9^ *k*-mers, which is at the same time complete and highly diverse.

The phage display-generated library provides a rich source of mimotopes that can be screened for different theranostic tools focused on particular targets. On an omics scale, the smaller optimized mimotope library proposed here probes the repertoire of broadly expressed IgM reactivities efficiently, mapping its dynamic diversity to a space of potentially over 10^70^ distinct profiles. The major tasks ahead are (1) exploring the concept of reproducibility for the sequences of IgM mimotopes by further deep panning experiments and (2) designing studies aimed at efficiently extracting specific diagnostic profiles and building appropriate predictors, for example, for predicting immunotherapy responders or side effects and predicting the risk of malignancy in chronic inflammation as well as other conditions involving immune activity.

## Data Availability Statement

The datasets generated for this study can be found in the GITHUB (https://github.com/ansts/IgMimoPap1 and https://github.com/ansts/IgMimoPap2).

## Ethics Statement

The studies involving human participants were reviewed and approved by Ethics Committee at the Medical University Sofia. The patients/participants provided their written informed consent to participate in this study.

## Author Contributions

AP conceptualized the project, analyzed the results performing all the *in silico* work, supervised experiments except for the sequencing as well as the overall project execution, and prepared the manuscript. MH ran the phage display experiments. VK and MT ran the microarray experiments up to data processing and cataloged and maintained the seroteque. VS participated in the conceptualizing of the paper and the overall design of the experiments, supervised the phage display experiments, and together with K-MH and LM-Z carried out the DNA isolation, PCR, and sequencing. EH supervised the sequencing task and participated in conceptualizing the project and the preparation of the manuscript. SP and MT performed the data processing of microarray scans. TV and TK-E participated in conceptualizing the project, analysis of the results, and the preparation of the manuscript. DF was responsible for the patient selection, informed consent, ethics committee protocol preparation, blood collection, and serum preparation.

### Conflict of Interest

The authors declare that the research was conducted in the absence of any commercial or financial relationships that could be construed as a potential conflict of interest.
