# Supplementary material for: Diagnostic Profiling of the Human Public IgM Repertoire With Scalable Mimotope Libraries
Source: Front Immunol. 2019 Dec 3;10:2796. doi: 10.3389/fimmu.2019.02796 (PMC6901697; doi:10.3389/fimmu.2019.02796)
Supplement: Supplementary file 1 [file Data_Sheet_1.pdf]

Supplemental Figures

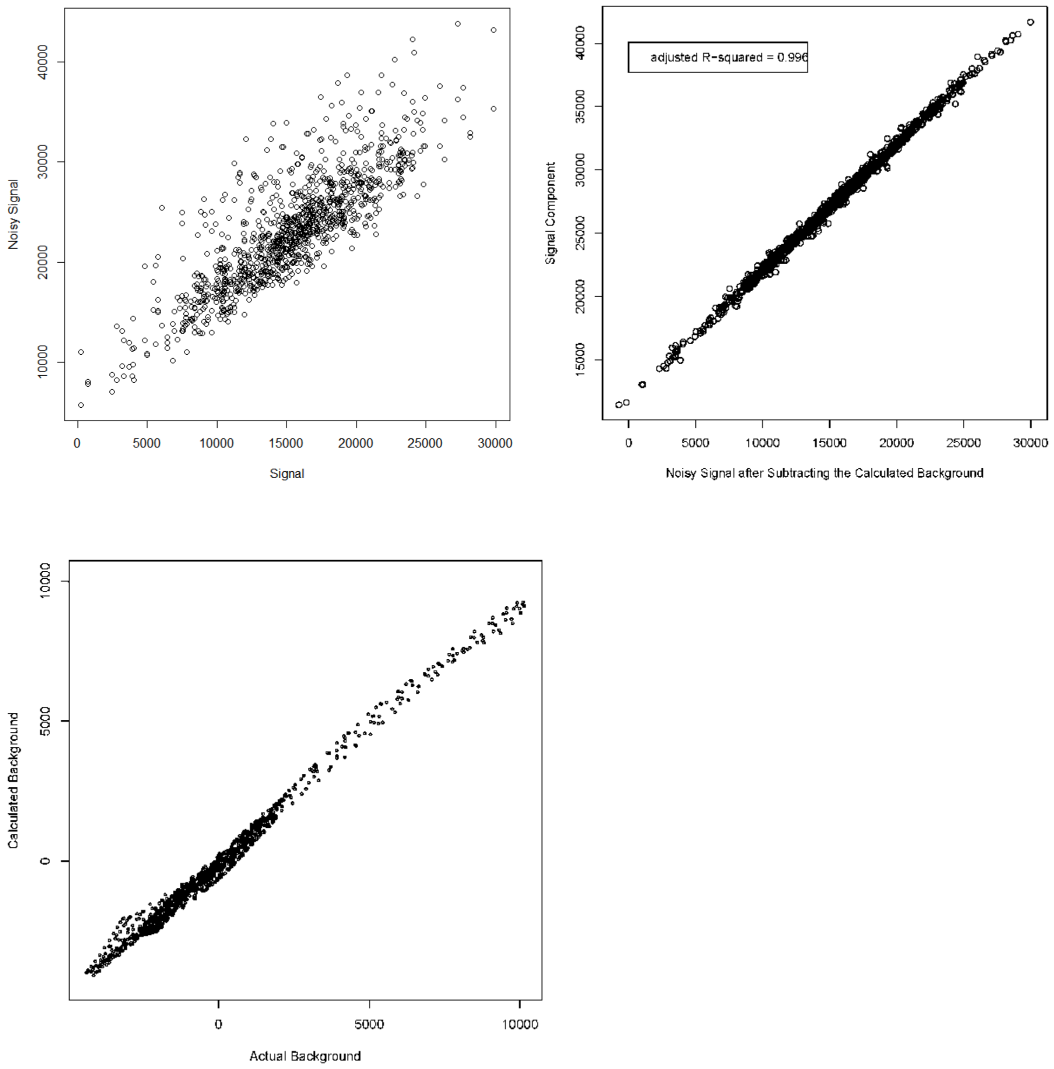

Suppl. Figure 1. Results of a simulated example of background subtraction for microarray data local normalization using the randomly positioned spot duplicates.

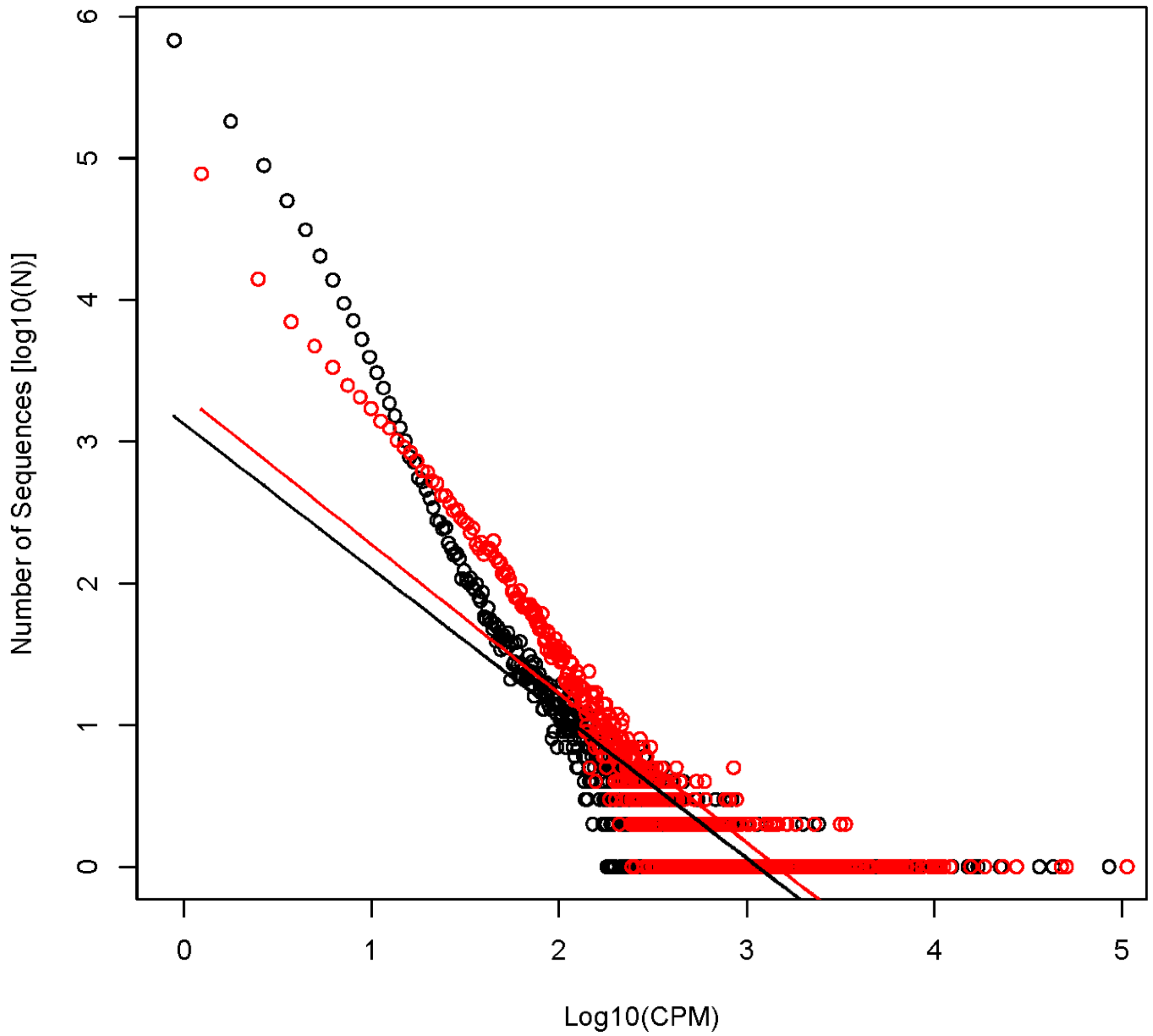

Suppl. Figure 2. Results from NGS. Plot of reads distribution by CPM (counts per million). Black – sample 1 – selected from original library, Red – sample 2 – selected form a pre-amplified original library. Here the selection caused deviation from the power law (the straight lines) with an emphasis on the lower CPM. The pre-amplification enriches in better fit phages and leads to a considerable shrinkage of the highly diverse compartment of low CPM where most of the targeted diversity lies.

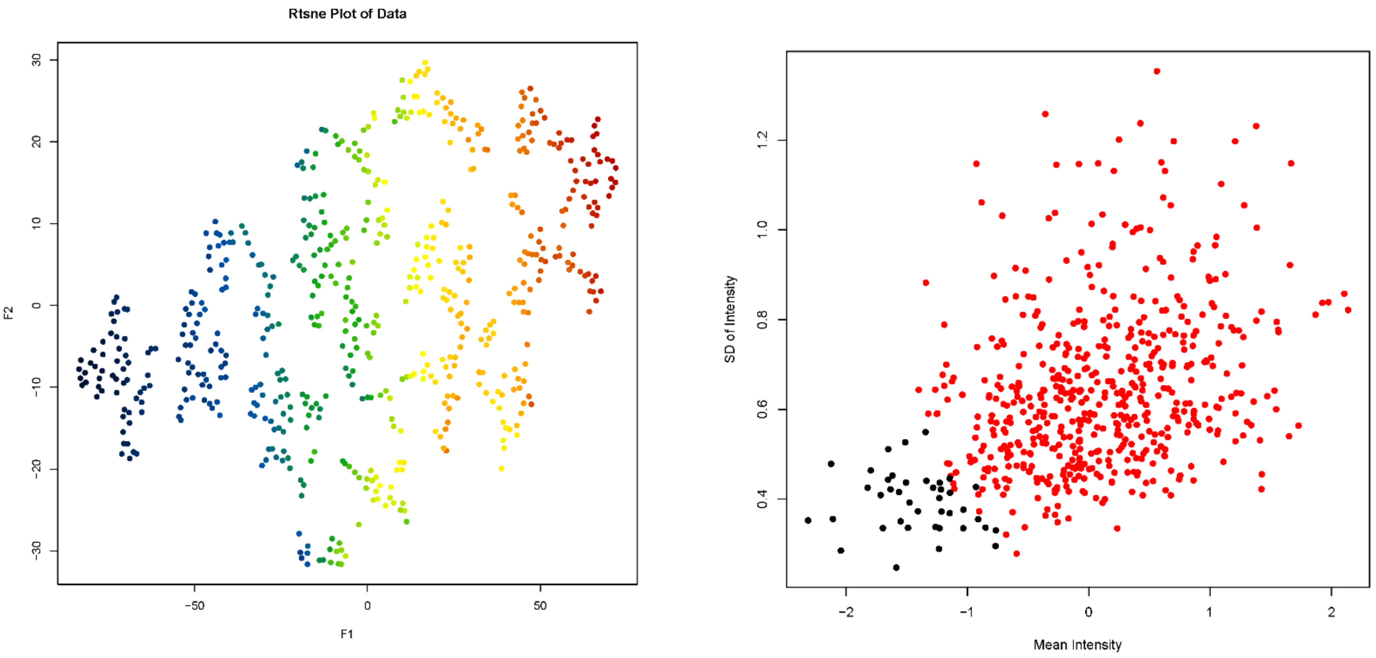

Suppl. Figure 3. Defining background binding by clustering using t-sne. Each point represents a single peptide profile formed by the reactivity with IgM from 10 patents' sera. The clustering separated a group of low binding peptides (left panel - color coded for mean binding, the low binding peptides considered negative are in black). Right panel – the actual SD by mean plot of the same data, black – the peptides delineated in the left panel with black color, red – the rest considered positively binding.

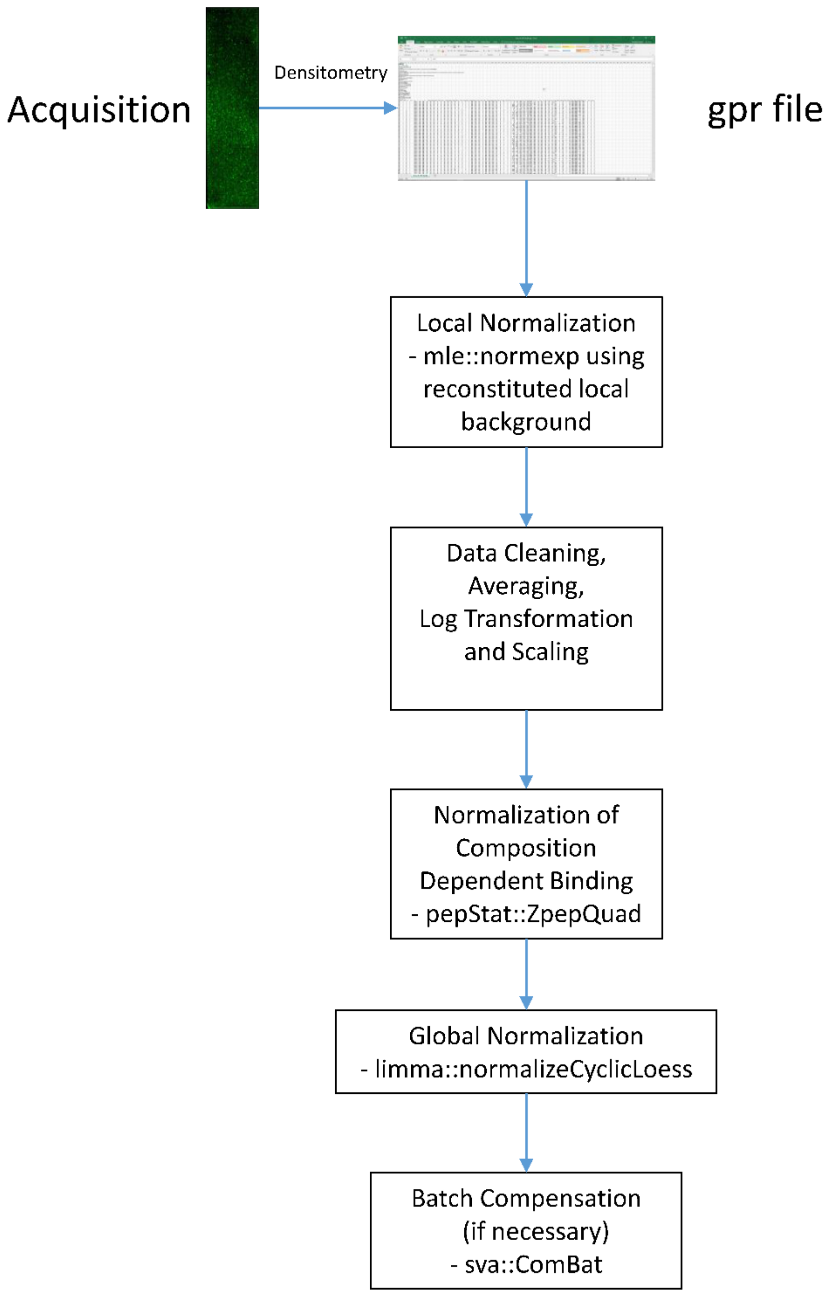

Suppl.Figure 4. Workflow for the microarray analysis.

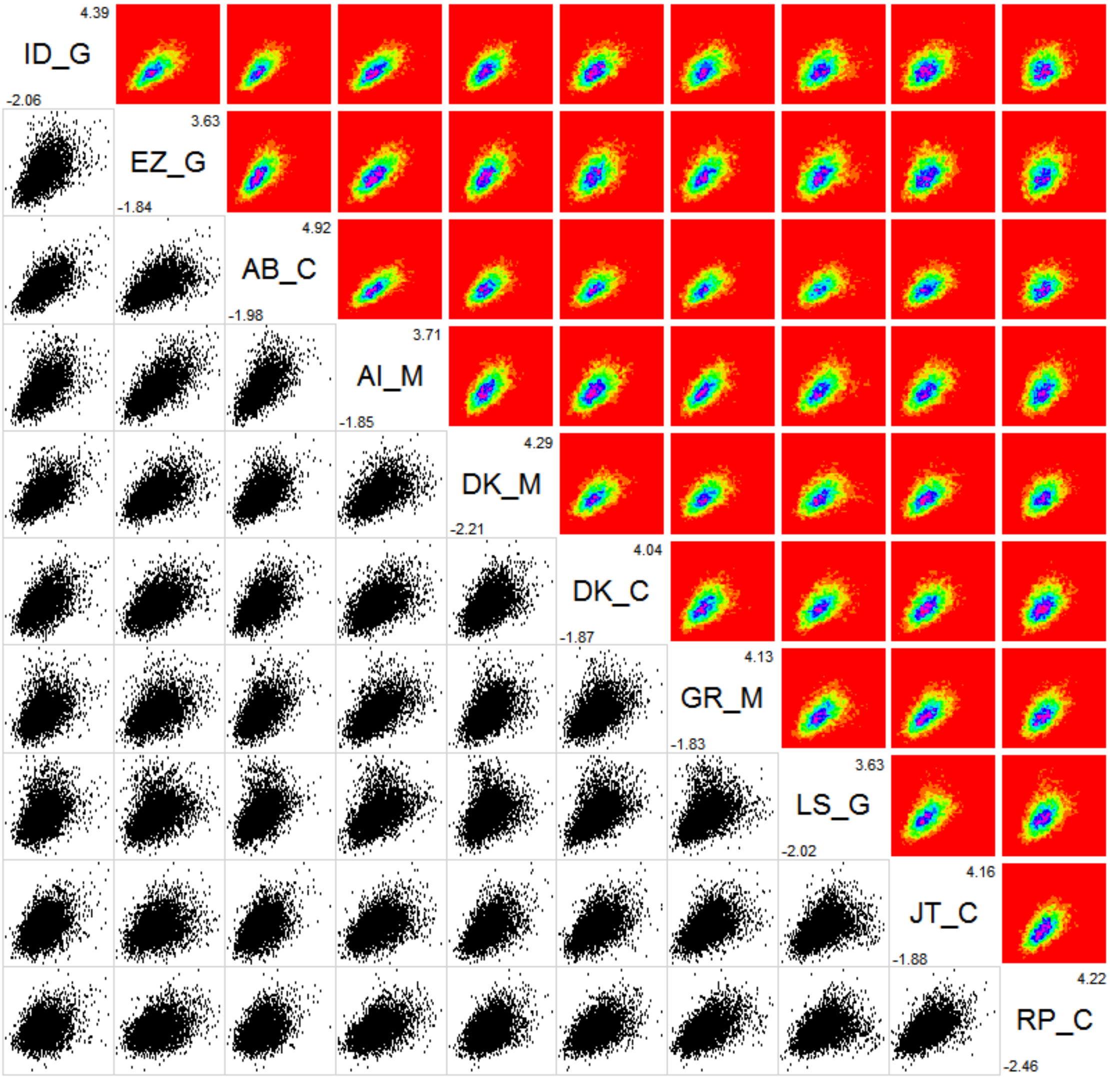

Suppl. Figure 5. Correlogram of data from the binding of patient serum IgM to a collection of 5500 peptides representing all the studied libraries. The data is log transformed and scaled and is plotted after local and global normalization.

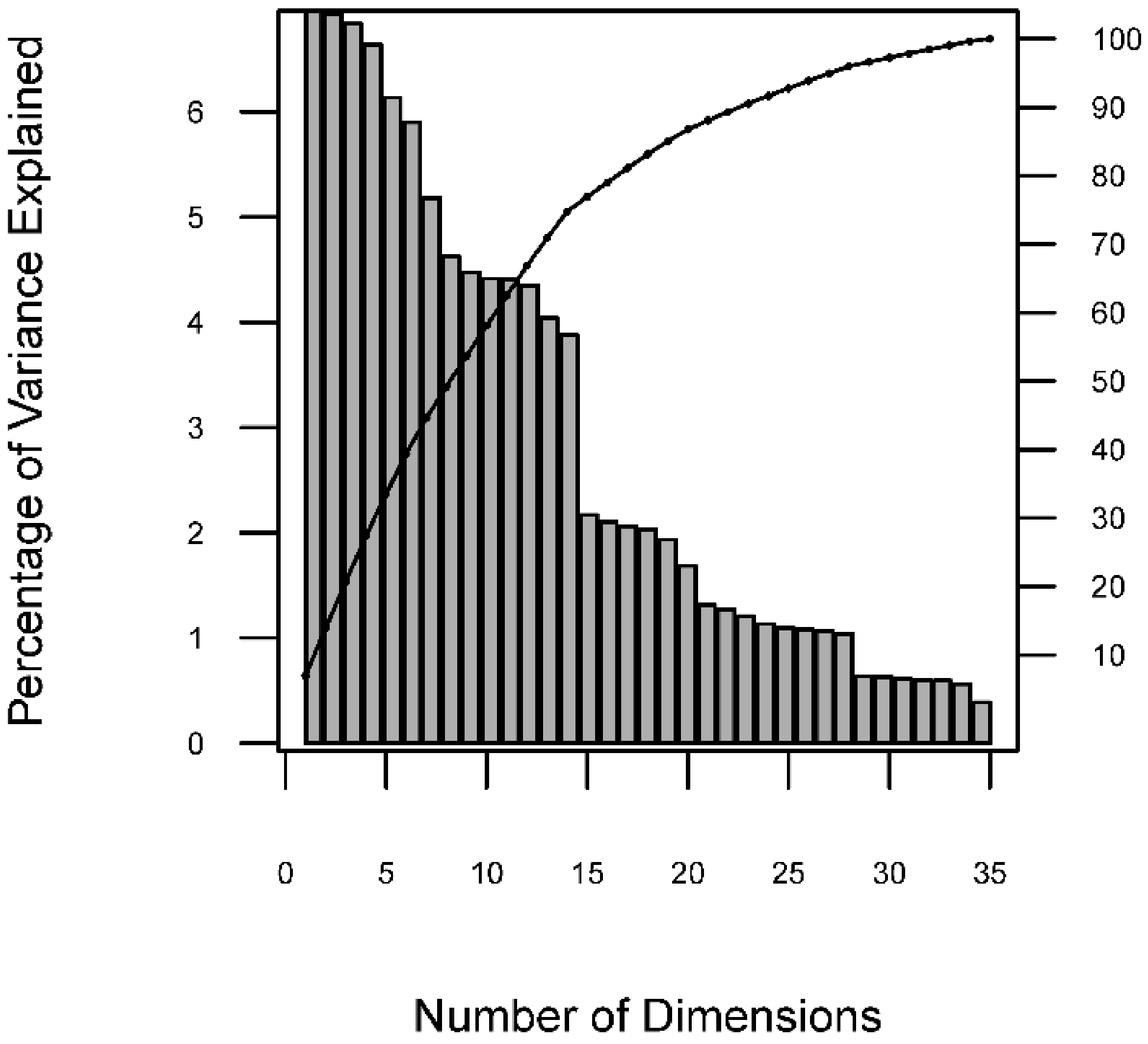

Suppl. Figure 6. Scree plot for the PCA on the phage selected mimotope library encoded using the five dimensional z-scores as described in Materials and Methods. The cumulative variance plot is associated with the right axis.

A

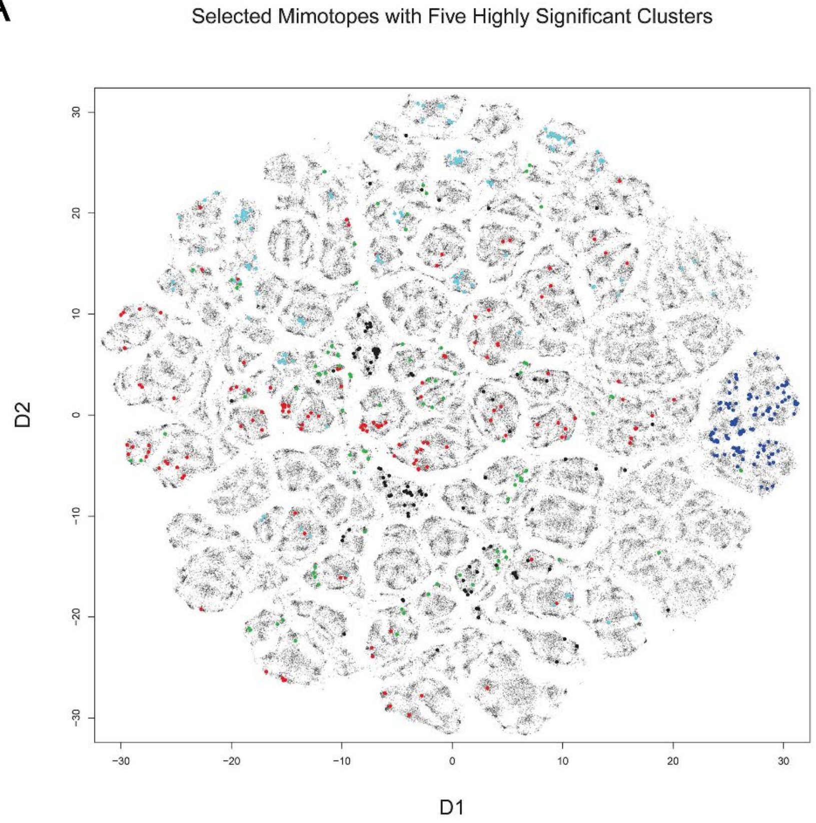

Selected Mimotopes with Five Highly Significant Clusters
D2
D1
30
20
10
0
−10
−20
−30

B

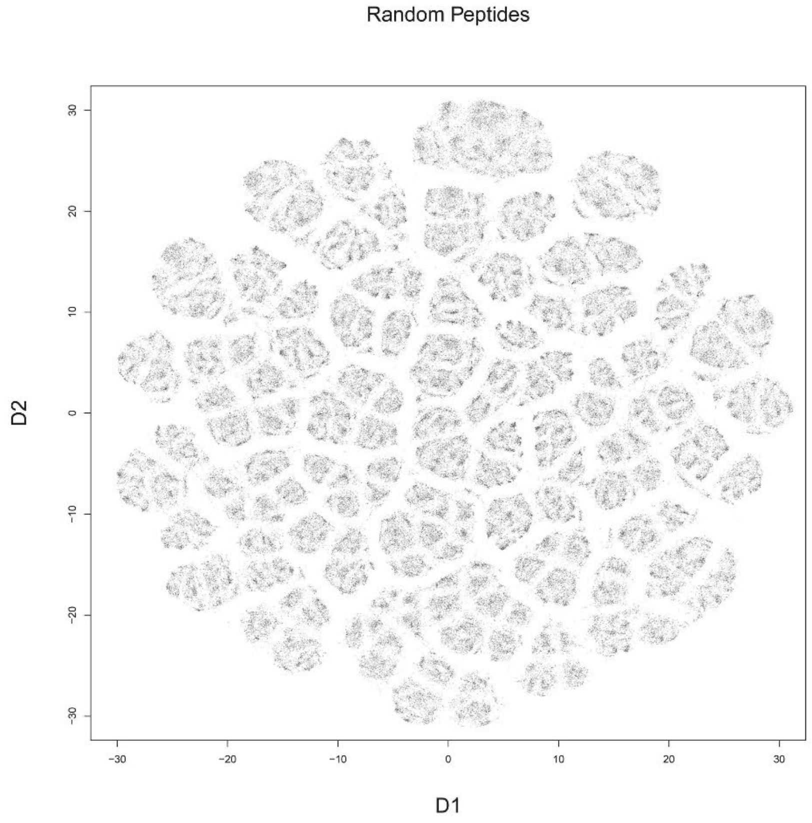

Random Peptides
D2
D1
30
20
10
0
−10
−20
−30

Suppl. Figure 7. Visualization of the 7-mer sequence space of the phage display selected mimotopes (A) and equal number random sequence with the same background residue frequencies (B) constructed using t-sne based on the Barnes-Hut algorithm. The peptides in five clusters (included in library pep5) are color coded in (A). The peptide sequences were encoded using a 5-dimensional score reflecting basic biophysical properties of the amino acids. Each sequence is represented by a 35 dimensional vector but the t-sne mapping is performed after initial reduction to 14 dimensions by PCA. There is only moderate correlation between the clustering visualized by t-sne and the GibbsCluster classification. The image is of high resolution and can be zoomed for better detail inspection.

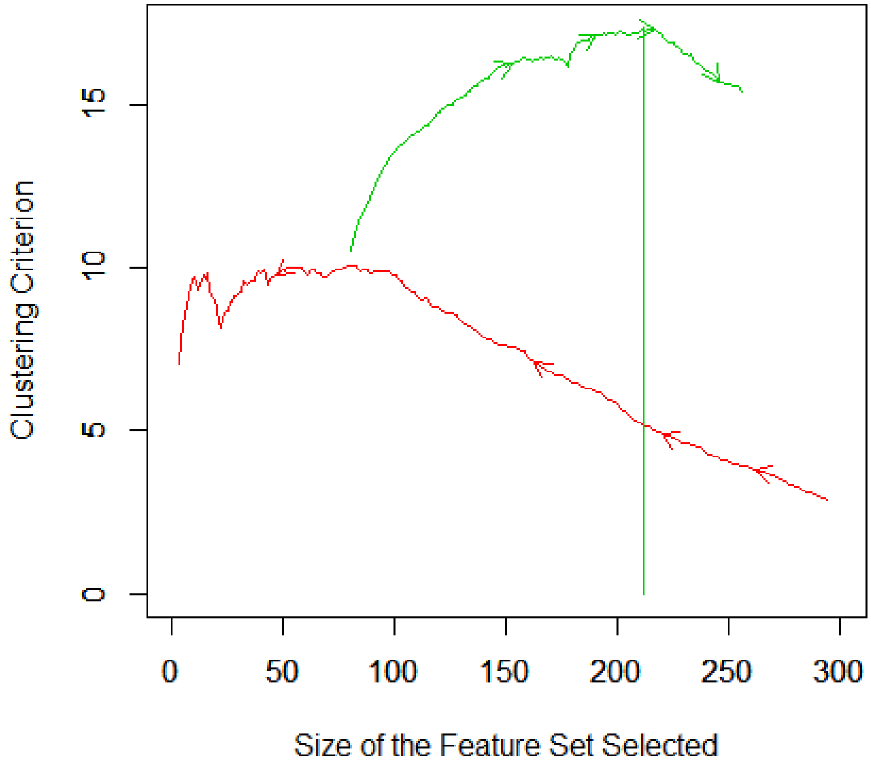

Suppl. Figure 8. A plot illustrating the backward/forward feature selection algorithms used. The search starts with the top 294 significantly expressed features and successively eliminates at each step the feature the removal of which results in the greatest increase of the clustering criterion (the process proceeds from the far end back to less features indicated by the arrows, red curve). After an optimal set of features is found (at the maximum of the red curve) the further elimination of even the most unnecessary feature leads to a fall in the criterion value. Thus, the feature set corresponding to the maximum of the lower curve is taken as the optimal one for this first stage of recursive feature elimination. In the next step of forward selection, starting with this optimal set, features which are not already included are added one at a time so that each step ensures maximal criterion increase. The maximum of this second curve (green vertical line) corresponds to the optimal set.

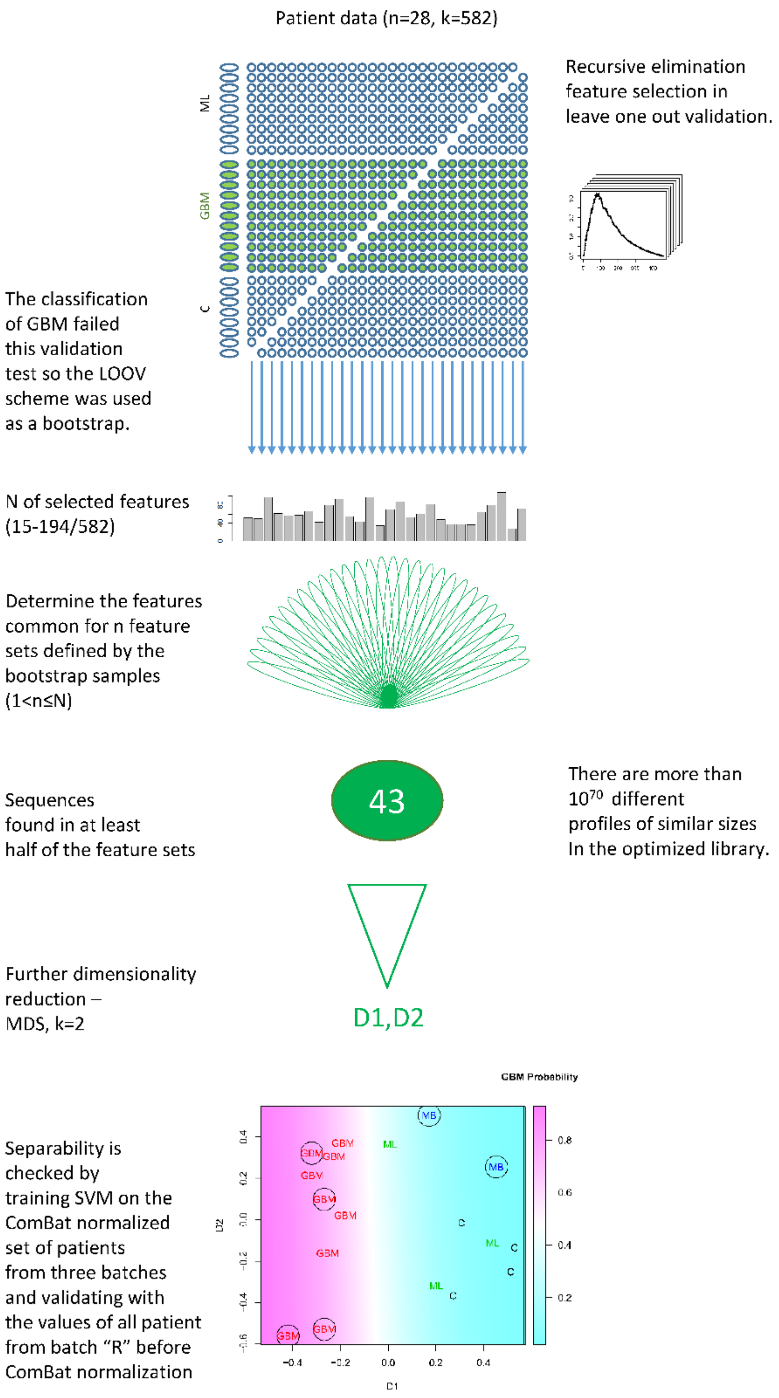

Suppl. Figure 9. Schematic representation of the feature selection scheme for extracting diagnostic profiles and a SVM model.
