# Supplementary material for: Diagnostic Profiling of the Human Public IgM Repertoire With Scalable Mimotope Libraries
Source: Front Immunol. 2019 Dec 3;10:2796. doi: 10.3389/fimmu.2019.02796 (PMC6901697; doi:10.3389/fimmu.2019.02796)
Supplement: Supplementary file 2 [file Data_Sheet_2.PDF]

Supplemental Methods

Microarray local normalization

Due to the dense microarray layout making impossible the local measurement of background intensities the latter were estimated. Support vector regression filters were used to that end. The estimated background levels were passed then to the R functions makePeptideSet from the pepStat package which uses backgroundCorrect from the package limma and the normexp algorithm to subtract background. For the initial analysis comparing different libraries, the approximation was based on the spots with intensities in the lower tertile. The tertile threshold is determined locally in overlapping patches of 5x5 spots. For the rest of the chips, the background approximation was done using the following method described below. It is based on the randomly positioned duplicates followed by a smoothing step using support vector regression.

By definition the duplicate spots P_i' and P_i'' contain the same peptide p_i. When stained under the same conditions they should have staining intensities I_i' and I_i'' each one being the sum of the same specific signal intensity S_i and most often different local background levels B_i' and B_i'' because they are positioned randomly on the chip.

$$I_i' = S_i + B_i' + e_i',$$
$$I_i'' = S_i + B_i'' + e_i'' \qquad (1)$$

Thus, the difference between the intensities of the duplicate spots of a given peptide p_i is independent of the peptide's signal intensity S_i but equals the difference of the background signal intensities ΔB_i between the two duplicate spots of peptide *i* plus an error term *e*:

$$\Delta I_i = I_i' - I_i'' = B_i' - B_i'' = \Delta B_i + e_i \qquad (2)$$

The accepted approach for determining the local background intensity for a spot is to measure it in an area adjacent to that spot when possible. This approach is based on the assumption that the local background is a smooth function of the position changing on a distance scale commeasurable with the dimensions of

the chip. The same assumption is still justified in the case of the peptide chips used in the current study even though the measuring of the local background is impossible. Thus, the spots on the chip with x-coordinate equal to a-1, a, or a+1, and y-coordinate equal to b-1, b, or b+1 for some a, b being closest to the spot $P_{a,b}$ are expected to have all very similar background levels. Their respective duplicates P_i'' $(i = 1:9)$ being randomly positioned most often lie away from the patch and, thus, usually have different background. Therefore, in a given patch of adjacent spots the average of the intensity differences between each spot $P'_{a,b}$ and its respective duplicate P_i'' are given by:

$$\frac{\sum_{i=a-1}^{a+1}\sum_{j=b-1}^{b+1}\Delta I_{i,j}}{9} = \frac{\sum_{i=a-1}^{a+1}\sum_{j=b-1}^{b+1}B'_{i,j} - \sum_{i=1:9}B_i''}{9} + \overline{\Delta e_{a-1..a+1,b-1..b+1}} \approx \overline{B'_{a-1..a+1,b-1..b+1}} - \overline{B_i''} \qquad (3)$$

where $\overline{B'_{a-1..a+1,b-1..b+1}}$ is the mean of the background values in the patch approximating the background at $B'_{a,b}$ and $\overline{B_i''}$ is the mean of the background levels of the randomly positioned duplicates of the spots in the patch. This makes sense because the mean of Δe by definition is approximately 0. Since the spots' duplicates of the spots in the patch are randomly scattered on the chip their mean $\overline{B_i''}$ can be used to approximate the global mean of the background of the chip. Thus, the mean of $\Delta I_{a,b}$ approximates the local background $B_{a,b}$ at position {a,b} centered to the global mean of the background of the chip. To improve the approximation, the calculated background values at each spot $P_{a,b}$ were iteratively adjusted by:

$$B_{a,b}^{j+1} = B_{a,b}^{j} - (\Delta B_{a,b}^{j} - \Delta I_{a,b}^{m})/s. \qquad (4)$$

Here $\Delta B_{x,y}^{j}$ is the difference in the estimated background level between the spot $P_{x,y}$ and its duplicate at step *j*, $\Delta I_{a,b}^{m}$ is the respective measured difference between spot $P_{a,b}$ and its duplicate and s is a factor empirically adjusted to 6 (Suppl. Fig. 1). The correlation between the original and the reconstructed signal in a simulated chip image was R^2=0.99. The background approximation, thus derived, is made completely independent of the signal by taking the differences between duplicates.

Mimotope selection

The reads from the deep sequencing experiment were processed using the script provided by Matochko et al. [1]. The Phred quality score cut off used was 32 with a probability for an erroneous base call of $6x10^{-4}$. The unique reads from experiments A and B were pooled so that only one copy of a sequence existed in the final set (n=1 100 124). These were further sorted based on the number of occurrences of each sequence. The criterion for retaining a sequence was an occurrence in 3-10 CPM, yielding 224 087 unique sequences using the following rationale. Considering that 78% of the single base changes lead to a change in the encoded amino acid [2] (see also phD7GC script for the mean substitution rate in Ph.D-7 library), the Phred score used led to a frequency of f=0.01 for the sequences with one erroneous amino acid residue if there is only one wrong base call per sequence. The occurrence of two or more errors in the same read is in the order of $f^2=10^{-4}$ and will be considered negligible. The probability for 3 reads with identical sequences to be simultaneously erroneous each at a single base is less than $f^3=10^{-6}$. If at least one of the reads is correct the presence of wrong calls in the triplet is non-consequential for the purpose of the mimotope library. Thus, the error rate of the mimotope calls with depth 3 at Phred score of 32 is expected to have produced no erroneous sequences.

The limit of 10 copies on the high copy numbers was applied after comparison of the distributions of the number of clones by CPM between the original and the preamplified library (Suppl. Fig. 2). It showed that the threshold of 10 copies was discriminating the original and preamplified libraries, with diversity in the latter skewed towards highly proliferating clones. This fact was interpreted in view of the observation that the affinity selection seemed to favor low CPM clones. Therefore, the high CPM clones were excluded to avoid a possible contamination with non-selected clones having an advantage when they are highly proliferating. This restriction led to the exclusion of 9.96% of the reads. Reads found in 3-10 copies were selected yielding 224087 sequences which contained none of the parasitic sequences reported by Matochko et al. [3]. This mimotope library was further subjected to clustering using the GibbsCluster-2.0

method [4]. The number of clusters was optimized using the Kulback-Leibler distance (KLD) from the background model of random sequences [4]. Position weighted matrices (PWM) were defined for each cluster using pseudo counts as follows:

$$PWM_{k,j} = log_2\left(\frac{\sum I\left(X_{i,j} = k\right) + b_k\sqrt{N}}{(N + \sqrt{N})b_k}\right)$$

where i=1..N are the rows of the alignment, j=1:7 are the columns of the alignment, I(aa=k) is the indicator function used to count the occurrences of amino acid k in column j and b_k is the background frequency of amino acid k in the phage display library [5].

Using the PWMs, the median of the log odds (LO) scores of the peptides in each cluster was calculated. This value can be seen as a score measuring the relevance of the sequence to the cluster. Next, a set of random sequences of 7 aa 10-fold larger than the analyzed library was generated in silico. The probability of the occurrence of such a random aa sequence with LO greater than the respective median of each cluster was determined empirically with the help of the generated random set. Using this estimate, the probability of the chance occurrence of as many peptides with scores higher than the median score as observed in each cluster was calculated using the binomial distribution. The probabilities, thus found for each cluster, were used as a criterion for their relevance to the mimotope library being generated.

For removing from random peptide sequences those that are related to the mimotope library, each of 2.3×10^6 random 7-mer peptide sequences was tested against each of the PWM of mimotope clusters defined. For each random peptide, only the score of the top scoring cluster was retained, and the peptides were ranked in the ascending order of these scores. The lowest ranking peptides represented random sequences that were the least related to any of the clusters in the selected library were used. This set can be considered purged of “mimotope like” sequences.

The clustering of the mimotopes was visualized using the t-sne (t-Distributed Stochastic Neighbor Embedding) algorithm [6] in its implementation using the faster Barnes-Hut algorithm (Rtsne package) with theta parameter default value of 0.5 and a maximum of 1500 iterations [7].

The amino acid residues were described using the 5-dimensional scale of amino acid residue properties published by Hellberg et al. [8]. The amino acid residue properties quantification used was the same as for the pepStat binding normalization [9]. The 5 scales (z1-z5) were extracted as the latent variables describing the major factors underlying the variability of amino acids in the space of 26 physicochemical parameters. Thus, each peptide was represented by a vector of 35 scores corresponding to its seven positions. For a comparison, the same number of random 7-mer peptides generated using the amino acid background frequencies of Ph.D.-7 were clustered using the same parameters. The clusters in the t-sne plot were labeled using k-mean clustering.

Microarray data treatment

Local normalization

The spot intensities of a non-treated chip were subtracted from the stained chip spot intensities. Treatment with secondary antibody only did not result in any binding. Next, the local background had to be inferred due to the dense spot layout. To that end an approximated background was smoothed by support vector regression and the result passed to the backgroundCorrect [10] function of the limma R package for local normalization using the normexp (mle) method. The initial background approximation is described above.

Global normalization

The peptides with missing values (flagged "bad") in some of the patients were removed. Log transformed locally normalized microarray data were next normalized for amino acid composition dependent binding using the ZpepQuad method of pepStat package [11]. This step is considered indispensable because of the strong effect of amino acid composition on binding due mostly to electrostatic interactions. The amino

acid residue properties were quantified using the 5 dimensional descriptor z1-z5 of Sandberg et al. (1998) [9]. This was followed by global normalization using normalizeCyclicLoess (method affy) from the package limma [10] and subjected to batch effect compensation using the ComBat [12] function from package sva whenever necessary (for different chip batches and for different channels). The data was acquired in 2 different batches using the green (batch G) and the red channel (batch R). Eleven cases were part of an earlier experiment and that subset of the data could be used also in this assay (batch P). For batch effect compensation the groups were balanced by selecting a subset of the patients with relatively even representation of each tested diagnosis. The criterion for inclusion of the 5 GBM patients from batch "R" was the minimal difference of the mean and coefficient of variation from the mean and CV of the whole group of GBM in that batch. Finally, each peptide binding intensity was represented by the mean of its duplicates.

Because of a lack of a suitable negative control, the baseline binding was determined from the data using the following approach. The cleaned data used for the testing of the diagnostic potential was subjected to dimensionality reduction of the peptide reactivities using t-sne [6], which clearly outlined a group of peptides with uniformly low reactivities, (Suppl. Fig. 3) that was considered background binding. The mean of the background binding intensity was subtracted from the data before testing for significant reactivities.

Library comparison

A general linear model, followed by Tukey contrast, was used to compare the mean expression of reactivities in the different tested libraries.

The optimal sampling of the mimotope reactivity space (projection of each peptide to the reactivity with 10 patients' serum IgM) was first measured by the mean nearest neighbor distance (NND) per library which was normalized (nNND) relative to the theoretical mean distance between the points in the 10-dimensional data cloud for the different libraries:

$$nNND_L = \frac{NND_L}{\left(\frac{V}{N_L}\right)^{\frac{1}{k}}},$$

where L=1..8 indicates the libraries, N_L is the number of peptides in library L, k is the dimensionality (10 since the peptides are compared on the basis to their reactivity to 10 patients' sera) and V is the volume of the data cloud approximated as a k-dimensional ellipsoid [13]:

$$V = \frac{2\pi^{\frac{k}{2}}}{k\Gamma\left(\frac{k}{2}\right)}\prod_{i=1}^{k}(2 * \sigma_i),$$

where σ_i is the standard deviation of the data along the i_{th} dimension (from the values of the i_{th} serum) and Γ is the gamma function. The logarithms of nNND were compared by a linear model.

It was interesting to check for the correlation between the patients' profiles with the peptide sets form each library. The mean correlation coefficient between all pairs of patient IgM reactivity profiles was used to that end. The greater the information content about each patient in the library profile the lower the probability that some patients will have correlated profiles. Linear models were used to compare the mean values of the mean correlation, thus calculated for the different libraries, after the correlation values were converted to z-scores.

Visualization of the Mimotope Space

T distributed stochastic neighbor embedding (t-sne) based on the Barnes-Hut algorithm was used to visualize the structure of the mimotope sequence space as represented by the general mimotope library produced by deep panning. The sequences were represented by converting each amino acid residue to a 5-dimensional vector of physical property scores as described in the Microarray data treatment section. Thus, each 7-mer peptide is represented by a 35-dimensional vector. Principle component analysis indicated that the first 14 principle components account for approximately 75% of its variance (Supplemental Figure 6). Therefore, the t-sne mapping was done after reducing the 35 dimensions to 14 by PCA (the initial.dims parameter of the Rtsne function of the R package with the same name).

Feature selection algorithm

The composition of the library underwent a small change - 75 of the lowest scoring sequences from NGR library were added to the library as a negative control. Because of the size limit of the final library, this addition was done by replacement of 75 of the selected positive peptides. Ultimately, these "negative" peptides had a lower proportion of reactive features - 49/206 non-significant and 24/380 significant reactivities ($\chi 2$, $p<0.0001$). The finding of individuals with IgM reactive for some of the NGR peptides is not a surprise. That is why the background reactivity was considered more reliably determined by the data analysis, rather than on the mean level of the NGR library.

For the design of a practical algorithm for extracting information about a particular diagnosis, a combination of filtering and wrapping feature selection techniques was applied next. The filtering method used was selection of individual features with highly significant expression in at least one patient. The top table of the mimotopes with significant reactivity with the separate patient's IgM produced by the limma package yielded arrays of mimotope sequences ordered by significance in descendent order. This data was presented as a matrix of mimotope ranks (rows - the mimotopes in descending order of significance and columns – the ranks of these mimotopes relative to each patients). The rank matrix was than reordered by the median rank or each row so that the mimotopes ranking higher in more patients were closer to the top.

The wrapping techniques were recursive feature elimination followed by a forward selection algorithm. The quality of clustering of the predetermine diagnostic groups was used as a criterion. In the recursive elimination, at each step the algorithm eliminates the feature the removal of which produces the greatest increase in the clustering criterion. This leads to an improvement of the clustering to a point at which the remaining features ensure optimal clustering. After that point the removal even of the feature that is least appropriate still leads to a decrease in the criterion. In the forward selection, similarly, to a preselected

set new features are added successively based on the same criteria. The cluster separation of the cases of interest was measured using a combined clustering criterion $Crit$:

$$B = BH\gamma(M,c), C = Conn(M,c), D = Dunn(M,c)$$

$$Bn = bhgamfix(B,\mathrm{d}), Cn = -connfix(C,d), Dn = dunnfix(D,\mathrm{d})$$

$$Crit = b^{Bn} + b^{Cn} + b^{Dn}$$

Where Dunn(M,c) is a function calculating the Dunn's clustering criterion [14,15] which is based on single inter-cluster and intra-cluster distances (extreme case), BHγ(M,c) calculates the Baker-Hubert Gamma index [16] which gives the overall agreement of distances and cluster assignment based on all the data, Conn(M,c) calculates the connectivity validation measure for a given clustering partitioning based on 10 nearest neighbors , which emphasizes the agreement of distances and partitions in the vicinity of each point; *M* is the matrix of distances between the cases, and *c* is the diagnosis code for the cases. All three criteria depend on the dimensionality which makes it difficult to compare across different dimensionalities. Therefore, they are normalized by the corresponding functions dunnfix(), confix() and bhgamfix() so that they no longer depend on the dimension of the data and change within the interval (-1, 1). Connectivity is then taken with an opposite sign so that its direction of change with improvement of clustering matches that of the other two criteria. Finally, the composite criterion equals the sum of the exponential functions of the criteria with a common base b. In this way the criteria are converted to non-negative values. The base b is picked randomly between 1.5 and 5 and stays the same for one run of the feature selection function. Switching the base between runs of the recursive elimination or forward feature selection leads to the selection of slightly different sets of features. Thus, by running several iterations of the recursive and forward selection (n=5) the search of the huge space of feature combinations is more effective. At the end of n iterations, the algorithm outputs the consensus profile – those features that were selected in all iterations.

Supplementary Figure 8 shows the typical traces of the clustering criterion as a function of the number of features remaining with the best set of features corresponding to the maximum of the clustering criterion. The recursive elimination features selection was performed on the features of significant expression in any patient (n=582).

The features optimal for a given group of patients varied greatly even when the patient groups differed by only 2 individuals (as tested in a leave one out scheme). Still, many features recurred in the sets derived from different patient groups. The common features were pooled based on the number of groups they were common for, i.e. – the feature set labeled 1 included all features, set n included features found in at least n patient groups and set 28 contained only the feature found in all groups (n=1). These sets were used to find the pooling level providing the best performing feature set.

To construct relevant models, the dimensionality of the reactivity data was reduced to two by MDS. The models were based on a radial basis function (Gaussian) kernel-based support vector machine (SVM) and was used to classify both the training set and the validation set of the cases of batch “R” omitted before the batch compensation. By including cases omitted before the batch compensation, the validation set served also as a control for the lack of confounding effect of the batch compensation. The performance of the model was measured using the Matthew’s correlation coefficient (MCC). Figure 7 shows that among the SVM models constructed on the bases of the different feature sets those based on sets found at least in 8,9,10,11,12,13,14,15 or 16 sets predicted perfectly the validation set. Although most of the models were much better at predicting the training set the one based on features found in at least 16 sets had a lower MCC. Thus, the best model with the least number of parameters remained the one based on features found in at least 15 sets. This is the one we used further (the number of features was 43). The significance of the MCC values was determined by comparing to the 5% and the 95% confidence limits of MCC calculated on models based on 1000 scrambled data matrices. The Supplemental Figure 9 shows a schematic representation of the used feature selection algorithm.

References to the Supplemental Methods

1. Matochko WL, Chu K, Jin B, Lee SW, Whitesides GM, Derda R (2012) Deep sequencing analysis of phage libraries using Illumina platform. Methods 58: 47-55.
2. Abdullah T, Faiza M, Pant P, Rayyan Akhtar M, Pant P (2016) An Analysis of Single Nucleotide Substitution in Genetic Codons - Probabilities and Outcomes. Bioinformation 12: 98-104.
3. Matochko WL, Cory Li S, Tang SK, Derda R (2014) Prospective identification of parasitic sequences in phage display screens. Nucleic Acids Res 42: 1784-1798.
4. Andreatta M, Lund O, Nielsen M (2013) Simultaneous alignment and clustering of peptide data using a Gibbs sampling approach. Bioinformatics 29: 8-14.
5. de Hoon M, Vitkup D (2006) Comparative Systems Biology of the Sporulation Initiation Network in Prokaryotes. In: Eskin E, Ideker T, Raphael B, Workman C, editors. Systems Biology and Regulatory Genomics: Joint Annual RECOMB 2005 Satellite Workshops on Systems Biology and on Regulatory Genomics, San Diego, CA, USA; December 2-4, 2005, Revised Selected Papers. Berlin, Heidelberg: Springer Berlin Heidelberg. pp. 62-69.
6. van der Maaten LJP, Hinton G (2008) Visualizing Data using t-SNE. Journ Machine Learning Res 9: 27.
7. van der Maaten L (2014) Accelerating t-SNE using Tree-Based Algorithms. J Machine Learn Res 15: 3221-3245.
8. Hellberg S, Sjoestroem M, Skagerberg B, Wold S (1987) Peptide quantitative structure-activity relationships, a multivariate approach. Journal of Medicinal Chemistry 30: 1126-1135.
9. Sandberg M, Eriksson L, Jonsson J, Sjostrom M, Wold S (1998) New chemical descriptors relevant for the design of biologically active peptides. A multivariate characterization of 87 amino acids. J Med Chem 41: 2481-2491.
10. Ritchie ME, Phipson B, Wu D, Hu Y, Law CW, Shi W, et al. (2015) limma powers differential expression analyses for RNA-sequencing and microarray studies. Nucleic Acids Res 43: e47.
11. Imholte G, Sauteraud R, Gottardo R (2016) Analyzing Peptide Microarray Data with the R pepStat Package. Methods Mol Biol 1352: 127-142.
12. Johnson WE, Li C, Rabinovic A (2007) Adjusting batch effects in microarray expression data using empirical Bayes methods. Biostatistics 8: 118-127.
13. Wilson JA (2010) Volume of n-dimensional ellipsoid. Sciencia Acta Xaveriana 1: 101-106.
14. Handl J, Knowles J, Kell DB (2005) Computational cluster validation in post-genomic data analysis. Bioinformatics 21: 3201-3212.
15. Dunn JC (1974) Well-Separated Clusters and Optimal Fuzzy Partitions. Journal of Cybernetics 4: 95-104.
16. Baker FB, Hubert LJ (1975) Measuring the Power of Hierarchical Cluster Analysis. Journal of the American Statistical Association 70: 31-38.
