# Supplementary material for: Diagnostic Profiling of the Human Public IgM Repertoire With Scalable Mimotope Libraries
Source: Front Immunol. 2019 Dec 3;10:2796. doi: 10.3389/fimmu.2019.02796 (PMC6901697; doi:10.3389/fimmu.2019.02796)
Supplement: Supplementary file 3 [file Data_Sheet_3.pdf]

Supplement Tables

Suppl. Table 1. General linear model results from comparison between mean peptide reactivity in different libraries (for library designation see Table 1).

Simultaneous Tests for General Linear Hypotheses

Multiple Comparisons of Means: Tukey Contrasts

Linear Hypotheses:	Estimate	Std. Error	z value	Pr(>|z|)	
pep5pred - pep5 == 0	0.040685	0.036587	1.112	0.9502	
pepneg - pep5 == 0	-0.101131	0.039385	-2.568	0.1563	
pepneglo - pep5 == 0	-0.001002	0.075444	-0.013	1	
pepnegrnd - pep5 == 0	-0.390134	0.036239	-10.766	< 0.0010	***
pepother5 - pep5 == 0	0.059812	0.03348	1.786	0.6110	
peppos - pep5 == 0	0.081682	0.03848	2.123	0.3810	
peprnd - pep5 == 0	-0.115647	0.036324	-3.184	0.0286	*
pepneg - pep5pred == 0	-0.141816	0.037469	-3.785	0.0035	**
pepneglo - pep5pred == 0	-0.041687	0.074462	-0.56	0.9992	
pepnegrnd - pep5pred == 0	-0.430819	0.034148	-12.616	< 0.0010	***
pepother5 - pep5pred == 0	0.019127	0.031204	0.613	0.9985	
peppos - pep5pred == 0	0.040997	0.036517	1.123	0.9477	
peprnd - pep5pred == 0	-0.156332	0.034237	-4.566	< 0.0010	***
pepneglo - pepneg == 0	0.100129	0.075876	1.32	0.8832	
pepnegrnd - pepneg == 0	-0.289003	0.037129	-7.784	< 0.0010	***
pepother5 - pepneg == 0	0.160943	0.034442	4.673	< 0.0010	***
peppos - pepneg == 0	0.182813	0.03932	4.649	< 0.0010	***
peprnd - pepneg == 0	-0.014516	0.037212	-0.39	1	
pepnegrnd - pepneglo == 0	-0.389132	0.074292	-5.238	< 0.0010	***
pepother5 - pepneglo == 0	0.060814	0.072986	0.833	0.9903	
peppos - pepneglo == 0	0.082684	0.07541	1.096	0.9539	
peprnd - pepneglo == 0	-0.114645	0.074333	-1.542	0.7706	
pepother5 - pepnegrnd == 0	0.449946	0.030795	14.611	< 0.0010	***
peppos - pepnegrnd == 0	0.471816	0.036168	13.045	< 0.0010	***
peprnd - pepnegrnd == 0	0.274487	0.033865	8.105	< 0.0010	***
peppos - pepother5 == 0	0.02187	0.033404	0.655	0.9978	
peprnd - pepother5 == 0	-0.175459	0.030895	-5.679	< 0.0010	***
peprnd - peppos == 0	-0.197329	0.036253	-5.443	< 0.0010	***

Signif. codes: 0 ‘***’ 0.001 ‘**’ 0.01 ‘*’ 0.05 ‘.’ 0.1 ‘ ’ 1
(Adjusted p values reported -- single-step method)

Suppl. Table 2. GLM results from comparison of mean nearest neighbor distance between pepetide profiles in different libraries (for library designation see Table 1).

```
Simultaneous Tests for General Linear Hypotheses

Multiple Comparisons of Means: Tukey Contrasts


Fit: glm(formula = log(value) ~ L1, data = mc10clnndist)

Linear Hypotheses:
                             Estimate Std. Error z value Pr(>|z|)    
pep5pred - pep5 == 0       -0.0463163  0.0204399  -2.266  0.29596    
pepneg - pep5 == 0         -0.0314013  0.0220028  -1.427  0.83374    
pepneglo - pep5 == 0        0.2372490  0.0421481   5.629  < 0.001 ***
pepnegrnd - pep5 == 0      -0.0576799  0.0202455  -2.849  0.07630 .  
pepother5 - pep5 == 0      -0.0583229  0.0187041  -3.118  0.03474 *  
peppos - pep5 == 0          0.0708564  0.0214974   3.296  0.01993 *  
peprnd - pep5 == 0         -0.0260619  0.0202927  -1.284  0.89734    
pepneg - pep5pred == 0      0.0149150  0.0209328   0.713  0.99629    
pepneglo - pep5pred == 0    0.2835652  0.0415995   6.817  < 0.001 ***
pepnegrnd - pep5pred == 0  -0.0113636  0.0190771  -0.596  0.99882    
pepother5 - pep5pred == 0  -0.0120067  0.0174327  -0.689  0.99701    
peppos - pep5pred == 0      0.1171727  0.0204009   5.744  < 0.001 ***
peprnd - pep5pred == 0      0.0202544  0.0191272   1.059  0.96175    
pepneglo - pepneg == 0      0.2686503  0.0423893   6.338  < 0.001 ***
pepnegrnd - pepneg == 0    -0.0262786  0.0207429  -1.267  0.90381    
pepother5 - pepneg == 0    -0.0269216  0.0192415  -1.399  0.84765    
peppos - pepneg == 0        0.1022577  0.0219666   4.655  < 0.001 ***
peprnd - pepneg == 0        0.0053394  0.0207890   0.257  1.00000    
pepnegrnd - pepneglo == 0  -0.2949288  0.0415043  -7.106  < 0.001 ***
pepother5 - pepneglo == 0  -0.2955719  0.0407746  -7.249  < 0.001 ***
peppos - pepneglo == 0     -0.1663925  0.0421291  -3.950  0.00185 ** 
peprnd - pepneglo == 0     -0.2633108  0.0415273  -6.341  < 0.001 ***
pepother5 - pepnegrnd == 0 -0.0006431  0.0172043  -0.037  1.00000    
peppos - pepnegrnd == 0     0.1285363  0.0202060   6.361  < 0.001 ***
peprnd - pepnegrnd == 0     0.0316180  0.0189192   1.671  0.68953    
peppos - pepother5 == 0     0.1291794  0.0186614   6.922  < 0.001 ***
peprnd - pepother5 == 0     0.0322611  0.0172599   1.869  0.55321    
peprnd - peppos == 0       -0.0969183  0.0202533  -4.785  < 0.001 ***
---
Signif. codes:  0 ‘***’ 0.001 ‘**’ 0.01 ‘*’ 0.05 ‘.’ 0.1 ‘ ’ 1
(Adjusted p values reported -- single-step method)
```

Suppl. Table 3. GLM results from comparison between z-score transformed mean correlation between patient profiles in different libraries (for library designation see Table 1).

```
Simultaneous Tests for General Linear Hypotheses

Multiple Comparisons of Means: Tukey Contrasts


Fit: glm(formula = value ~ variable, data = corptzm)

Linear Hypotheses:
                            Estimate Std. Error z value Pr(>|z|)    
pep5pred - pep5 == 0        0.010342   0.027400   0.377  0.99995    
pepneg - pep5 == 0         -0.024782   0.027400  -0.904  0.98574    
pepneglo - pep5 == 0       -0.114978   0.027400  -4.196  < 0.001 ***
pepnegrnd - pep5 == 0      -0.059773   0.027400  -2.181  0.36269    
pepother5 - pep5 == 0      -0.003980   0.027400  -0.145  1.00000    
peppos - pep5 == 0         -0.133697   0.027400  -4.879  < 0.001 ***
peprnd - pep5 == 0         -0.051258   0.027400  -1.871  0.57126    
pepneg - pep5pred == 0     -0.035123   0.027400  -1.282  0.90583    
pepneglo - pep5pred == 0   -0.125319   0.027400  -4.574  < 0.001 ***
pepnegrnd - pep5pred == 0  -0.070115   0.027400  -2.559  0.17162    
pepother5 - pep5pred == 0  -0.014322   0.027400  -0.523  0.99955    
peppos - pep5pred == 0     -0.144039   0.027400  -5.257  < 0.001 ***
peprnd - pep5pred == 0     -0.061600   0.027400  -2.248  0.32313    
pepneglo - pepneg == 0     -0.090196   0.027400  -3.292  0.02225 *  
pepnegrnd - pepneg == 0    -0.034991   0.027400  -1.277  0.90753    
pepother5 - pepneg == 0     0.020801   0.027400   0.759  0.99505    
peppos - pepneg == 0       -0.108915   0.027400  -3.975  0.00182 ** 
peprnd - pepneg == 0       -0.026476   0.027400  -0.966  0.97906    
pepnegrnd - pepneglo == 0   0.055204   0.027400   2.015  0.47168    
pepother5 - pepneglo == 0   0.110997   0.027400   4.051  0.00127 ** 
peppos - pepneglo == 0     -0.018719   0.027400  -0.683  0.99744    
peprnd - pepneglo == 0      0.063720   0.027400   2.325  0.28004    
pepother5 - pepnegrnd == 0  0.055793   0.027400   2.036  0.45724    
peppos - pepnegrnd == 0    -0.073924   0.027400  -2.698  0.12324    
peprnd - pepnegrnd == 0     0.008515   0.027400   0.311  0.99999    
peppos - pepother5 == 0    -0.129717   0.027400  -4.734  < 0.001 ***
peprnd - pepother5 == 0    -0.047278   0.027400  -1.725  0.67064    
peprnd - peppos == 0        0.082439   0.027400   3.009  0.05339 .  
---
Signif. codes:  0 ‘***’ 0.001 ‘**’ 0.01 ‘*’ 0.05 ‘.’ 0.1 ‘ ’ 1
(Adjusted p values reported -- single-step method)
```
